# Exchange catalysis by tapasin exploits conserved and allele-specific features of MHC-I molecules

**DOI:** 10.1038/s41467-021-24401-4

**Published:** 2021-07-09

**Authors:** Huan Lan, Esam T. Abualrous, Jana Sticht, Laura Maria Arroyo Fernandez, Tamina Werk, Christoph Weise, Martin Ballaschk, Peter Schmieder, Bernhard Loll, Christian Freund

**Affiliations:** 1 Laboratory of Protein Biochemistry, Institute for Chemistry & Biochemistry, Freie Universität Berlin, Berlin, Germany; 2 Artificial Intelligence for the Sciences, Department of Mathematics and Computer Science, Freie Universität Berlin, Berlin, Germany; 3 Core Facility BioSupraMol, Institute for Chemistry & Biochemistry, Freie Universität Berlin, Berlin, Germany; 4 Leibniz-Forschungsinstitut für Molekulare Pharmakologie, Berlin, Germany; 5 Laboratory of Structural Biology, Institute for Chemistry & Biochemistry, Freie Universität Berlin, Berlin, Germany

**Keywords:** Enzyme mechanisms, NMR spectroscopy, Antigen presentation, MHC class I

## Abstract

The repertoire of peptides presented by major histocompatibility complex class I (MHC-I) molecules on the cell surface is tailored by the ER-resident peptide loading complex (PLC), which contains the exchange catalyst tapasin. Tapasin stabilizes MHC-I molecules and promotes the formation of stable peptide-MHC-I (pMHC-I) complexes that serve as T cell antigens. Exchange of suboptimal by high-affinity ligands is catalyzed by tapasin, but the underlying mechanism is still elusive. Here we analyze the tapasin-induced changes in MHC-I dynamics, and find the catalyst to exploit two essential features of MHC-I. First, tapasin recognizes a conserved allosteric site underneath the α_2-1_-helix of MHC-I, ‘loosening’ the MHC-I F-pocket region that accomodates the C-terminus of the peptide. Second, the scoop loop_11–20_ of tapasin relies on residue L18 to target the MHC-I F-pocket, enabling peptide exchange. Meanwhile, tapasin residue K16 plays an accessory role in catalysis of MHC-I allotypes bearing an acidic F-pocket. Thus, our results provide an explanation for the observed allele-specificity of catalyzed peptide exchange.

## Abstract (web summary)

Tapasin is part of the peptide loading complex necessary for presenting antigenic peptides on MHC-I for the induction of adaptive immunity. Here the authors show that tapasin interacts with MHC-I in both conserved and allele-specific regions to promote antigen presentation, with tapasin L18 and K16 residues both implicated in this molecular interaction.

## Introduction

Major Histocompatibility Complex class I (MHC-I) molecules present peptide antigens on the surface of nucleated cells for immune surveillance, allowing CD8^+^ cytotoxic T-lymphocytes to eliminate infected or cancerous cells^1^. Selective loading of peptides derived from the cellular proteome to nascent MHC-I molecules requires the action of the peptide loading complex (PLC) in the endoplasmic reticulum (ER), which consists of the chaperone tapasin, the oxidoreductase ERp57, calreticulin and the transporter associated with antigen processing (TAP)^2^. Tapasin, a key constituent of the PLC, bridges nascent MHC-I heterodimers with the peptide pool delivered by the TAP1/TAP2 heterodimer and catalyzes the peptide editing of MHC-I molecules^3–6^. TAPBPR (TAP-binding protein-related protein), a homolog of tapasin, can also catalyze the peptide editing of MHC-I molecules but independent of the PLC, thereby providing a second quality control step for efficient loading of antigen^7–14^.

The single-particle cryo-electron microscopy (cryo-EM) structure of the native PLC provides critical insights into the overall architecture of this molecular machinery and shows that the F-pocket region (α_2-1_-helix and β-strands 7-8) of the MHC-I peptide binding groove is clamped by the N-terminal domain of tapasin^15^. However, the “scoop loop” (loop_11–20_ connecting β-strands 1–2 of tapasin), which is hypothesized to interact with the critical F-pocket region of MHC-I, is neither resolved in this structure nor in the crystal structure of tapasin in complex with ERp57^15,16^. Crystal structures of the TAPBPR-MHC-I complex revealed that the corresponding “scoop loop” (loop_22-36_ connecting β-strands 1–2) of TAPBPR remodels the peptide binding groove of the MHC-I in the region of the α_2-1_ helix, thereby promoting peptide dissociation and subsequently stabilizing the peptide receptive conformation of MHC-I^17–20^. Furthermore, the TAPBPR scoop loop was also suggested to act as a kinetic trap that lowers the affinity requirements for peptide binding but also prevents dissociation of high-affinity ligands under conditions of limiting peptide^21^. Interestingly, the scoop loop_11–20_ in tapasin is considerably shorter compared to the corresponding loop in TAPBPR, leading to the question whether tapasin and TAPBPR engage similar or distinct molecular mechanisms to foster peptide exchange.

MHC-I proteins are highly polymorphic and display conformational dynamics in their peptide binding groove^22–26^, which confers differential dependence on tapasin or TAPBPR for efficient peptide editing and cell surface expression^5,8,27–34^. The F-pocket of MHC-I, which accommodates the C-terminus of the bound peptide, is critical for MHC-I stability and peptide binding and displays high allelic variation^35,36^. The leucine side chain of a murine tapasin scoop-loop-derived linear peptide was observed to anchor to the F-pocket of H2-D^b^ devoid of two C-terminal residues of the antigen^37^. This raises the question whether the interplay of the tapasin loop_11–20_ with MHC-I polymorphic regions relates to the observed allele-specific binding and catalysis of tapasin.

Here we investigate dynamic changes of MHC-I in the presence of tapasin by NMR spectroscopy and observe conformational dynamics affected by tapasin binding in two regions of the MHC-I molecule. We then address the role of individual residues of the loop_11–20_ for tapasin-catalyzed peptide exchange in vitro and define the critical role of tapasin residues L18 and K16 for tapasin allele-specific activity. Our results thus uncover an allele-specific function of the tapasin scoop loop_11–__20_ in antigen presentation.

## Results

### Dynamic changes of MHC-I upon shifting into a peptide-receptive state

**Fig. 1:**
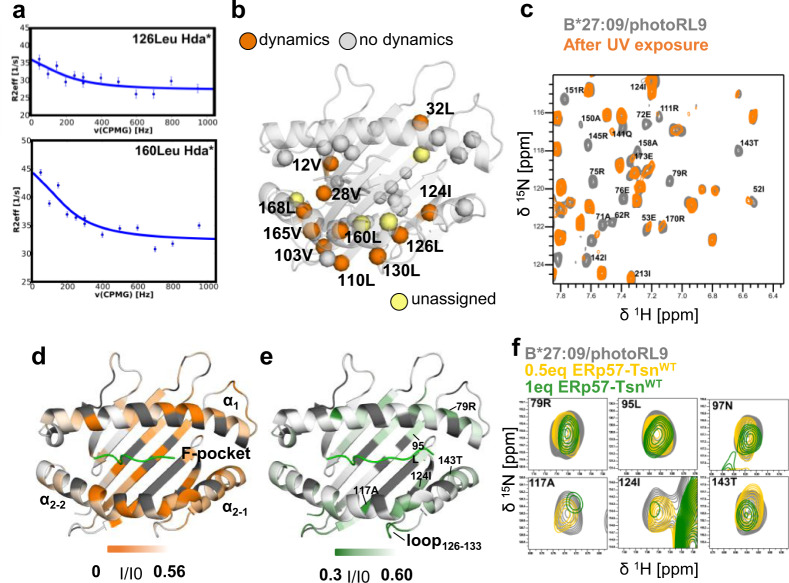
Tapasin “loosens” C-terminus of bound peptide in the pMHC-I. **a** Representative relaxation dispersion curves of B*27:09/photoRL9 methyl groups undergoing conformational exchange (${R}_{2}^{{eff}}\,$> 2 s^−1^) in ^1^H-^13^C methyl-CPMG experiments recorded at 27 °C at a ^1^H field of 700 MHz. The effective transverse relaxation rate (${R}_{2}^{{eff}}$) is shown as a function of the CPMG pulse frequency (ν_CPMG_) for selected methyl groups. Errors were estimated based on duplicate measurements. **b** Residues showing μs-ms dynamics derived from methyl-CPMG experiments of B27:09/photoRL9 (170 μM) are highlighted as orange spheres in the structure of B*27:09. Other assigned ILV methyl residues which did not show dynamics are mapped as gray spheres. Unassigned residues are shown in light yellow. **c** Zoom view of ^1^H-^15^N TROSY-HSQC spectra of B*27:09/photoRL9 before (gray) and after UV exposure (orange). Peaks vanished upon UV exposure are labeled. **d** Peak intensity ratio (*I*_uv-__exposed_/*I*_no-uv_) of cleaved B*27:09/photoRL9 (*I*_uv-exposed_) relative to non-cleaved B*27:09/photoRL9 (I_no-uv_) is mapped as B-factor in the structure of B*27:09 heavy chain. The range of the intensity ratio is from 0 (orange) to the average I_uv-exposed_/I_no-uv_ (0.58, white). Unassigned residues are colored in dark gray. **e** Peak intensity ratio (I/I0) of B*27:09/photoRL9 in the presence of ERp57-Tsn^WT^ (I) relative to B*27:09/photoRL9 alone (I0) is mapped as B-factor in the structure of B*27:09 heavy chain. The range of the intensity ratio is from half (0.30, forest) to the average I/I0 (0.6, white). Unassigned residues are colored in dark gray. **f** Representative peaks of residues lining the F-pocket showing reduced signal in the presence of ERp57-Tsn^WT^ (0.5eq in yellow, 1eq in forest).

To better understand the peptide-editing process of MHC-I, we investigated the dynamic properties of MHC-I in complex with a photocleavable peptide (pMHC-I), and then followed the changes upon UV exposure by NMR spectrometry. First, we characterized the dynamics of the human allotype B*27:09 in complex with the photoRL9 peptide (peptide sequence is listed in Supplementary Table 1) by ^1^H-^13^C multiple-quantum Carr-Purcell-Meiboom-Gill (CPMG) relaxation dispersion NMR experiments. The methyl groups of isoleucine, leucine and valine residues in the MHC-I heavy chain were ^1^H-^13^C-labeled. This method allows to detect conformational exchange of the main conformation with sparsely populated states on the micro- to milliseconds (µs-ms) timescale. Indeed, we could observe a relatively large number of methyl-group containing residues exerting conformational exchange and these residues are mainly in the binding groove and polarized towards the α_2_-helix side of it (Fig. 1a, b). Subsequently we followed the dynamics features of the complex after peptide cleavage. UV exposure of the photoRL9 peptide results in a 7-mer and a dipeptide fragment. The melting temperature *T*_m_ of the cleaved B*27:09/photoRL9 was lower than that of the intact pMHC-I (Supplementary Table 2). Mass spectrometry analysis showed that the 7-mer fragment remains bound to the binding groove of B*27:09 after UV exposure (Supplementary Fig. 1a). The ^1^H-^15^N TROSY-HSQC spectrum of cleaved B*27:09/photoRL9 was recorded and compared with that of the non-cleaved B*27:09/photoRL9 (Supplementary Fig. 1b).

Upon UV exposure, resonances of residues in the binding groove underwent either (i) chemical shift changes (ii) line broadening that is indicative of dynamics in the time range of µs-ms or (iii) both (Fig. 1c, d and Supplementary Fig. 1b). Chemical shift changes, which indicate an altered chemical environment due to the dissociation of the C-terminal dipeptide or rearrangement of residues that constitute the peptide and MHC-I interface, were observed throughout the binding groove (Supplementary Fig. 1c, upper chart). Line-broadened resonances, which indicate altered dynamics, were observed throughout the binding groove as well (Supplementary Fig. 1c, lower chart). In particular, residues which are part of the architecture of the F-pocket, including T143, which forms a hydrogen bond with the C-terminus of the peptide, residues L95, N97, and A117 at the bottom of the F-pocket of MHC-I as well as residues R75, E76, D77, and R79, from the α_1_-helix in the F-pocket region, showed strong line broadening (Fig. 1c), indicating altered dynamics of this region upon UV exposure. In addition to the F-pocket region, heavy chain residues 151–155 that are located close to the central part of the peptide, are also strongly affected, indicating that the altered dynamics extended towards the middle of the binding groove. In contrast, none of the assigned residues in the α_3_ domain showed significant signal reduction or chemical shift changes (Supplementary Fig. 1c).

Furthermore, line-broadened peaks re-appeared in the spectrum when the UV-cleaved B*27:09/photoRL9 was incubated with the intact RL9 peptide and the recovered spectrum looks the same as the ^1^H-^15^N TROSY-HSQC spectrum of B*27:09/RL9 indicating MHC-I molecules to reversibly adopt a peptide-receptive conformation upon UV exposure. Thus, our data indicates that the release of the C-terminal dipeptide fragment in the F-pocket upon UV exposure turns B*27:09/photoRL9 into a peptide-receptive state, featured by structural and dynamic changes in the binding groove, especially changes in the F-pocket region of the pMHC-I.

### Interaction with tapasin promotes pMHC-I to adopt a peptide-receptive state

We then aime at revealing structural and dynamic changes in pMHC-I complexes induced in the presence of the peptide editor tapasin. Since tapasin alone to enhance peptide loading shows low efficiency^4,38,39^, tapasin was co-expressed with ERp57 as a conjugate to maintain its full function^4^. Studies showed peptide exchange can be enhanced in vitro by ERp57-Tsn conjugate^16,40^, or by Tsn when linked to the heavy chain to increase the affinity^6^. In our system, ERp57-Tapasin conjugate (wild-type, referred to as ERp57-Tsn^WT^) can catalyze peptide exchange of non-cleaved B*27:09/photoRL9, although slowly (Supplementary Fig. 2a). Thus, we added stoichiometric amount of ERp57-Tsn^WT^ to intact B*27:09/photoRL9, to mimic the interaction between tapasin and suboptimal-peptide-loaded-MHC-I within the PLC and subsequently recorded the ^1^H-^15^N TROSY-HSQC spectrum.

While almost no chemical shift changes could be seen (Supplementary Fig. 2b), line-broadened resonances of residues in the peptide binding groove were observed (Fig. 1e, Supplementary Fig. 2b). Interestingly, many of these resonances (e.g. R79, N97, A117, D120, I124, T143, A151, Fig. 1f) overlapped with those affected by UV exposure (compare Fig. 1d, e and Supplementary Fig. 2b), although not as strongly as in the UV exposed spectrum. In particular, signal intensity of F-pocket residue T143 is significantly attenuated, together with other F-pocket residues N97, A117 (Fig. 1f), indicating that tapasin-induced altered F-pocket dynamics similar to the features seen for the pMHC-I molecule devoid of the C-terminal dipeptide after UV- exposure. Thus, we suggest that tapasin promotes pMHC-I to adopt a receptive state by affecting the residues in the F-pocket that interact with the C-terminus of the peptide, facilitating release of bound peptide and eventually exchanged by an incoming competitor peptide.

In addition to the F-pocket region, residues in the loop_126-133_ connecting strands β7-β8, were also significantly affected by addition of ERp57-Tsn^WT^ (Supplementary Fig. 2d), which is in line with the observed interaction between this region with the hairpin loop_187–__196_ (loop connecting β-strands 9–10) of tapasin in the cryo-EM structure of the PLC (Supplementary Fig. 2c)^15^. Notably, I124, L126 and L130, close or within the loop_126-133_ that was affected by tapasin binding, also showed conformational exchange dynamics in the intact pMHC-I complex (Fig. 1b), suggesting that an alternative conformation of this loop might pre-exist in solution and could be recognized by tapasin. Indeed, mutations of residues (D128, W133) within the loop_126-133_ have been previously shown to affect the MHC-I interaction with tapasin and it was suggested that the conformation of the loop_126-133_ might be modulated by tapasin^41^. Thus, the loop_126-133_ represents an important region for complex formation.

There are also some important differences between tapasin-induced and UV-exposure-induced changes in MHC-I. While individual residues of the F-pocket clearly indicate changes at the site of interaction with the C-terminus of the peptide in both cases, a full destabilization of the α_2-1_-helix is only observed after UV exposure.

### Influence of ERp57-Tsn on peptide exchange kinetics

Although the peptide exchange of un-cleaved B*27:09/photoRL9 can be enhanced in the presence of ERp57-Tsn^WT^ (Supplementary Fig. 2a), the association rate was extremely slow, and we thus decided to perform subsequent experiments under conditions of UV exposure. To be able to draw more general conclusions, we performed our analysis with two allotypes, B*27:09 and B*27:05, which only differ at position 116 (H116 and D116, respectively), and two pre-bound photocleavable (photoRL9 and photoIF9) as well as two incoming peptides (FITC-RL9 and FITC-IF9). The photoRL9 has a significantly higher predicted affinity than photoIF9 for B*27:09 as well as B*27:05 (Supplementary Table 1). To indirectly obtain the affinities of the cleaved peptide for MHC-I variants we compared the thermostability of the four combinations of allotypes and pre-bound peptides after peptide cleavage. We observed a 6 and 2 °C higher stability of the B*27:09 and B*27:05 in complex with photoRL9 compared to photoIF9, respectively (Supplementary Fig. 3a). This indicates a correspondingly higher affinity of the cleaved photoRL9 (7-mer) to the complexes. Peptide exchange kinetics were then monitored by adding the fluorescent-labeled versions of the corresponding peptides directly after UV-cleavage, either in the absence or presence of equimolar ERp57-Tsn^WT^ (Supplementary Fig. 3b–e). The uncatalyzed peptide exchange rate (*K*_*on*_) was higher for the less stable cleaved photoIF9 complexes (Supplementary Fig. 3f) while the enhancements of the *K*_*on*_ by ERp57-Tsn^WT^ for both allotypes were larger in case the higher affinity peptide photoRL9 was used as pre-bound peptide (Supplementary Fig. 3g). In contrast, the affinity of the incoming peptide affects the enhancement of *K*_*on*_ by ERp57-Tsn^WT^ to a lower extend when the allotype is in complex with the same photocleavable peptide (Supplementary Fig. 3g). These results are in line with the previous finding obtained with the B*44:02 allotype that showed tapasin to catalyze thermodynamically favored but kinetically disfavored exchange of suboptimal peptides for high-affinity peptides^40^.

### Allotypic differences in tapasin-catalyzed peptide exchange in vitro

**Fig. 2:**
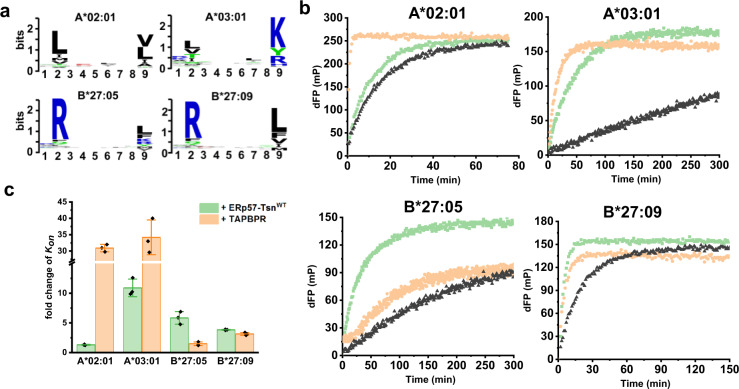
The susceptibility of MHC-I allotypes to tapasin-mediated peptide exchange is different. **a** Sequence logo of peptide that binds to A*02:01, A*03:01, B*27:05, and B*27:09. Peptides known to bind to these alleles were downloaded from IEDB and sequence logos were generated in https://weblogo.berkeley.edu. **b** Representative peptide exchange profiles of 500 nM A*02:01/photoKV9, A*03:01/photoKK9, B*27:05/photoRL9, B*27:09/photoRL9 in the absence (black) and presence of equimolar amounts of ERp57-Tsn^WT^ (light green) or TAPBPR (wheat) after UV exposure. Peptide exchange was monitored after adding the FITC-version of the peptide used in refolding for each allotype (FITC-GV9 for A*02:01, FITC-KK9 for A*03:01 and FITC-RL9 for B*27:09/05). The measuring time was different for each allotype. dFP means the baseline was subtracted. **c** The fold changes of observed association rate *K*_*on*_ in the presence of ERp57-Tsn^WT^ or TAPPBR for four allotypes. Error bars (standard deviation, SD) were calculated from three independent experiments.

It has been shown that the F-pocket region of pMHC-I is important for its tapasin dependence^32^. When we compared the tapasin-induced fold change of *K*_*on*_ of B*27:05 with B*27:09 using the same pre-bound and incoming peptides, B*27:05 is found to be more susceptible to tapasin-mediated peptide exchange than B*27:09 (Supplementary Fig. 3g). To delineate how the F-pocket architecture more generally correlates with its tapasin dependence we extended our analysis to two HLA-A allotypes. The allotype A*02:01 comprises a hydrophobic F-pocket with Y116 forming the floor while A*03:01 contains an Asp at this position, as it is in B*27:05. The charged surface area of the individual allotypes’ F-pocket is displayed in Supplementary Fig. 4a. Clearly, the F-pocket of these four allotypes shows a gradient in acidity: A*03:01 > B*27:05 > B*27:09 > A*02:01, in line with a differential preference for basic or hydrophobic residues at the C-terminus (PΩ) of bound peptides (Fig. 2a).

Peptide exchange experiments were performed by adding the FITC-version of the high-affinity peptides used in refolding for each allotype after UV exposure. The peptide affinity and thermal stability data of the corresponding photo-MHC-I molecules are listed in Supplementary Table 1 and 2, respectively. After UV exposure, in the presence of ERp57-Tsn^WT^, the peptide exchange rate was increased 10.89 ± 1.48 fold for A*03:01, 5.83 ± 1.06-fold for B*27:05, 3.83 ± 0.07-fold for B*27:09, 1.31 ± 0.08-fold for A*02:01 (Fig. 2c). ERp57-Tsn^WT^ slightly accelerates the peptide exchange of A*02:01 (Fig. 2b, c) and similar results were obtained for other high-affinity FITC-labeled peptides (Supplementary Fig. 4b, c), which is in agreement with previous studies showing that refolding of A*02:01 can be facilitated by tapasin^42^. Thus, our data indicate that susceptibility to tapasin-mediated peptide exchange of human allotypes appears to be positively correlated with the gradient of acidity of the MHC-I F-pocket.

We then generated F-pocket mutants of A*02:01, A*03:01, and B*27:05 to probe the positive correlation between tapasin enhancement on peptide exchange and the acidity of the F-pocket (F-pocket mutants are listed in Supplementary Table 3). Indeed, the peptide exchange rates of B*27:05H114R and B*27:05D116Y refolded with photoRL9 was less enhanced by tapasin than that of B*27:05WT (Supplementary Fig. 4d, e, the acidity of the F-pocket is: B*27:05WT > B*27:05D116Y > B*27:05H114R). A similar trend was observed for the F-pocket mutants of A*03:01 (Supplementary Fig. 4f–h). In this case the FITC-KK9 used for monitoring peptide association of A*03:01WT had to be replaced by FITC-KV9-2 (sequence is listed in Supplementary Table 3) for A*03:01D116Y and A*03:01R114H/D116Y mutants due to the low affinity of FITC-KK9 for these two mutants. A comparison of the peptide exchange of these two F-pocket mutants showed less tapasin dependence for A*03:01D116Y compared to that of A*03:01R114H/D116Y, which is in line with the acidity gradient of the mutants F-pocket (Supplementary Fig. 4f–h). In the case of the A*02:01 allotype, no significant tapasin enhancement was observed for the A*02:01Y116D mutant or the A*02:01H114D mutant (Supplementary Fig. 4i–k), indicating that the intrinsically low dependence on tapasin cannot be further enhanced by acidification of the F-pocket in this case.

In addition, we performed peptide exchange experiments with TAPBPR. In the presence of TAPBPR (Fig. 2b), peptide exchange rates of A*02:01, A*03:01, B*27:09, and B*27:05 were increased by 30.9 ± 1.12, 34.28 ± 5.3, 3.13 ± 0.23, and 1.48 ± 0.23-fold, respectively (Fig. 2c), which is in line with previous reports that TAPBPR has a strong preference for HLA-A alleles^28^. It is worth noting that in our experimental set up B*27:09 is more TAPBPR susceptible than B*27:05. This is in accord with a previous report where in the presence of TAPBPR no significant enhancement of peptide binding for B*27:05 was detected, while for B*27:09 an enhancement of more than 100% was observed^28^.

We then ask the question which structural or dynamic features of tapasin could explain the observed different susceptibility to tapasin-catalyzed peptide exchange for different allotypes.

### L18 is the key residue in the scoop loop_11–__20_ of tapasin to mediate peptide exchange of MHC-I

**Fig. 3:**
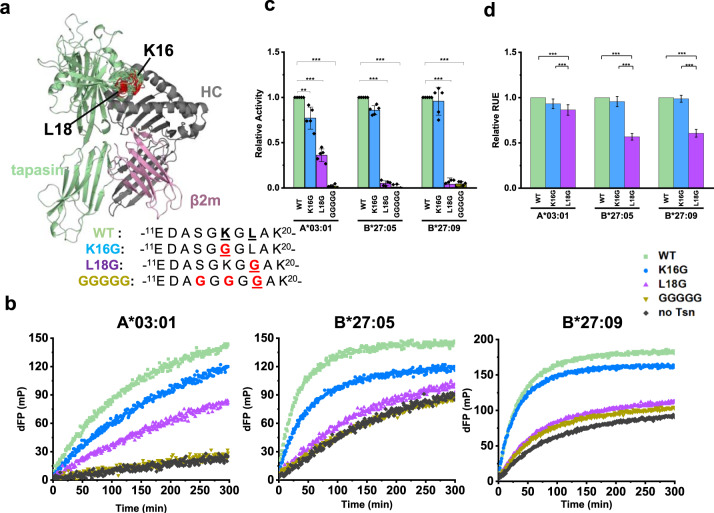
The role of tapasin scoop loop_11–__20_ in mediating peptide exchange of MHC-I. **a** The loop_11–__20_ of tapasin in the modeled MHCI-ERp57-Tsn complex (ERp57 is not shown here). Residue 16 and 18 were colored red. The sequence of tapasin scoop loop_11–__20_ mutants are shown besides. **b** Representative peptide exchange profiles of 500 nM A*03:01/photoKK9, B*27:05/photoRL9, B*27:09/photoRL9 in the absence (black) and presence of equimolar amounts of ERp57-Tsn^WT^ (light green), ERp57- Tsn^L18G^ (sky blue), ERp57-Tsn^K16G^ (purple), and ERp57-Tsn^GGGGG^ (golden). FITC-KK9 for A*03:01 and FITC-RL9 for B*27:05. FTIC-IF9 (IRAAK^FITC^PPLF) was used for better resolution of the kinetics for B*27:09. dFP means the baseline was subtracted. **c** Relative catalytic activity of ERp57-Tsn loop_11–__20_ mutants compared to ERp57-Tsn^WT^ towards three allotypes. The observed association rates (*K*_*on_Tsn_mutant*_) were determined for each mutant on the corresponding MHC-I allotype and relative activity was calculated by the formula of (*K*_*on_Tsn_mutant*_ – K_on_Tsn_^-^)/*(K*_*on_Tsn_WT*_ – *K*_*on_ Tsn*_^-^*)*. Error bar (SD) was calculated from five independent experiments. **d** Normalized free energy values of the modeled tapasin mutants calculated in Rosetta Energy Unit (REU). Error bar (SD) was calculated from 100 data points. In **c** comparison of loop mutants to WT, the two-sample unequal variance Student’s *t* test was performed, in **d** mean comparison method is Tukey, *p < 0.05, **p < 0.01, ***p < 0.001.

In the current cryo-EM PLC structure, residues within the tapasin scoop loop_11–20_ which are close to the F-pocket of MHC-I are not resolved, nor in the crystal structure of ERp57-Tsn conjugate alone, hinting at an increased flexibility of this region. We then used the cryo-EM structure of the PLC (PDB ID 6ENY) as a scaffold to model an ensemble of MHC-I-ERp57-Tsn complex structures to illustrate potential conformations of loop_11–20_ of tapasin (Fig. 3a). To test the potential function of this scoop loop for tapasin catalytic activity, we mutated the two amino acids K16 and L18 that have a side-chain conceivably long enough to reach into the F-pocket (Fig. 3a). Glycine was chosen as a substitute to allow for maximum flexibility of the loop, potentially allowing the residues at position 16 or 18 to reach into the F-pocket. As a control, the hypothetically fully inactive mutant ERp57-Tsn^GGGGG^ was also included. The stability of mutants was similar to ERp57-Tsn^WT^ (Supplementary Fig. 5). Since the peptide exchange of A*02:01/photoKV9 was only slightly enhanced by tapasin, we focused on A*03:01, B*27:05, and B*27:09 allotypes and investigated the catalytic activity of the mutants ERp57-Tsn^L18G^, ERp57-Tsn^K16G^ and ERp57-Tsn^GGGGG^ on peptide exchange. The relative catalytic activity of the mutants compared to the wild type were calculated by the formula *((K*_*on_Tsn_mutant*_ – K_on_Tsn_^-^)/(K_*on_Tsn_WT*_ – *K*_*on_ Tsn*_^-^*)* for each allotype.

As shown in Fig. 3b, the activity of ERp57-Tsn^L18G^ in catalyzing the peptide exchange of these three allotypes was strongly reduced (0.36 ± 0.06 for A*03:01, 0.05 ± 0.02 for B*27:05, and 0.04 ± 0.06 for B*27:09, Fig. 3c, purple bars), indicating that L18 is critical for tapasin catalytic activity. In contrast to that, the catalytic activity of ERp57-Tsn^K16G^ on peptide exchange was 0.77 ± 0.12 for A*03:01, 0.86 ± 0.05 for B*27:05, and 0.95 ± 0.15 for B*27:09 (Fig. 3c, blue bars), displaying a negative correlation to the gradient of acidity of the allotypes’ F-pocket. Expectedly, the catalytic activity of ERp57-Tsn^GGGGG^ on peptide exchange for all allotypes was completely abolished (0.01 ± 0.01 for A*03:01, 0.02 ± 0.01 for B*27:05 and 0.03 ± 0.02 for B*27:09, Fig. 3c, golden bars).

Taken together, for B*27:05 and B*27:09, the activity of ERp57-Tsn was nearly abolished by the L18G mutation, resulting in a peptide exchange rate nearly equal to that of uncatalyzed MHC-I (black curve, Fig. 3b), while the K16G mutation did not significantly impair the activity of ERp57-Tsn. Thus, for the B*27:05 and B*27:09 allotypes, L18 but not K16 appears to be important for tapasin catalytic activity. Different to B*27:05 and B*27:09, the peptide exchange of A*03:01 was still enhanced by ERp57-Tsn^L18G^ (Fig. 3b), indicating that K16 can rescue the activity of ERp57-Tsn^L18G^ for A*03:01 to a certain extent. Concomitantly, ERp57-Tsn^K16G^ showed significantly reduced activity (0.73 ± 0.12) for A*03:01, suggesting that K16 is important for the catalytic activity of tapasin in case of the A*03:01 allotype.

Additionally, we calculated the Rosetta Energy Scores (REU) of the MHC-I-ERp57-Tsn complex models with the desired scoop loop mutation for each allotype^43–45^. The REU were averaged for all generated models per mutant. As shown in Fig. 3d, the relative REU (rREU) of all three MHC-I allotypes in complex with ERp57-Tsn^K16G^ was only slightly reduced as compared to ERp57-Tsn^WT^ (0.93 ± 0.05 for A*03:01, 0.95 ± 0.05 for B*27:05, and 0.98 ± 0.03 for B*27:09), suggesting a marginal impact of the K16G mutation on overall complex stability. In contrast, rREU of the MHC-I -ERp57-Tsn^L18G^ complex was reduced to 0.56 for B*27:09 and 0.60 for B*27:05, indicating the important role of L18 to stabilize these complexes. Interestingly, rREU of A*03:01-ERp57-Tsn^L18G^ was still 0.86 that of wild type, again indicating that L18 is not the only important residue for stabilization of this allotype.

Taken together, our data highlight the critical role that L18 plays for tapasin catalytic activity while K16 plays an auxiliary role that is of importance for allotypes bearing an acidic F-pocket as exemplified here by A*03:01.

### The role of K16 in the loop_11–__20_ is dependent on the F-pocket nature of MHC-I molecules

**Fig. 4:**
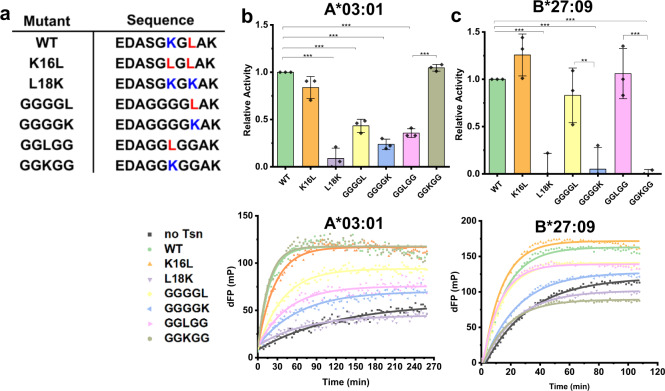
The role of K16 is the F-pocket nature dependent. **a** The sequence of Tsn loop_11–__20_ mutants**. b**, **c** upper panel: Relative activity of tapasin loop_11–__20_ mutants to catalyze peptide exchange compared to ERp57-Tsn^WT^ towards A*03:01 and B*27:09. FTIC-KK9 was used for A*03:01, FTIC-IF9 (IRAAK^FITC^PPLF) was used for better resolution of B*27:09. Error bars (SD) were calculated from three independent experiments. For comparison of loop mutants to WT, the two-sample unequal variance Student’s *t* test was performed (*p < 0.05, **p < 0.01, ***p < 0.001). Lower panel: Representative peptide exchange profiles of 500 nM A*03:01/photoKK9, and B*27:09/photoRL9 in the absence (black) and presence of equimolar amounts of tapasin loop mutants. dFP means the baseline was subtracted. These curves were recorded on the PerkinElmer VICTOR^3^V machine.

In order to unravel the relevance of the tapasin loop_11–20_ for the tapasin catalytic activity on MHC allotypes that differ in the chemical nature of their F-pocket, we designed several tapasin mutants with different combinations of leucine or lysine at positions 16 and 18 of the loop_11–__20_: K16L (SGLGL), L18K (SGKGK), GGGGL, GGGGK, GGLGG, and GGKGG (sequences are listed in Fig. 4a, stability data was listed in Supplementary Fig. 5). The A*03:01 allotype bearing the most acidic F-pocket (preference for basic residues) and B*27:09 bearing the least acidic F-pocket (preference for hydrophobic residues) were chosen to test the catalytic activity of these tapasin mutants.

As can be seen in Fig. 4b, c, the ERp57-Tsn^K16L^ mutant showed no significantly different catalytic activity compared to ERp57-Tsn^WT^ for A*03:01 (0.93 ± 0.16 of WT) and increased catalytic activity for B*27:09 (1.26 ± 0.06 of WT). While ERp57-Tsn^L18K^ displayed no activity for both allotypes. The relative activity of ERp57-Tsn^GGGGL^ was 0.40 ± 0.12 for A*03:01 and 0.77 ± 0.14 for B*27:09, while the relative catalytic activity of ERp57-Tsn^GGGGK^ was 0.23 ± 0.05 for A*03:01 and showed no catalytic activity for B*27:09. However, ERp57-Tsn^GGKGG^ and ERp57-Tsn^GGLGG^ showed an opposite behavior in catalysing peptide exchange of A*03:01 and B*27:09: ERp57-Tsn^GGKGG^ can enhance peptide exchange of A*03:01 as efficiently as ERp57-Tsn^WT^, while ERp57-Tsn^GGLGG^ can only slightly enhance the peptide exchange of A*03:01 (with 0.35 ± 0.05, the activity was similar to that observed for ERp57-Tsn^L18G^, 0.36 ± 0.05 of WT). In contrast, ERp57-Tsn^GGKGG^ was not able to promote peptide exchange of B*27:09 at all, while the activity of ERp57-Tsn^GGLGG^ on B*27:09 was the same as ERp57-Tsn^WT^. These results indicate that the exact position of K16 and critical L18 residues is malleable to a certain extent and the contribution of K16 is distinct for the two allotypes.

The catalytic activity of the ERp57-Tsn^K16L^ mutant, which has two leucine residues in the loop, was slightly higher than ERp57-Tsn^WT^ for B*27:09, suggesting position 16 can contribute to the activity of tapasin, again demonstrating that the role of the residue at position 16 strongly depends on the polarity of the F-pocket and a similar dichotomy was observed for position 18, albeit not as pronounced as for position 16. The opposite activity of ERp57-Tsn^GGKGG^ and ERp57-Tsn^GGLGG^ to catalyze peptide exchange of A*03:01 and B*27:09 suggests that when there is no Leu at position 18, only one Leu or Lys at position 16 in the loop is sufficient for tapasin to maintain its peptide exchange function. The activity of ERp57-Tsn^GGLGG^ was higher than that of ERp57-Tsn^GGGGL^ for B*27:09, demonstrating the importance of the loop’s mobility, indicating that the residue at position 16 can reach into the F-pocket better than Leu in position 18 in the context of a flexible loop.

### The interaction of ERp57-Tsn mutants with MHC-I indicates critical structural elements

**Fig. 5:**
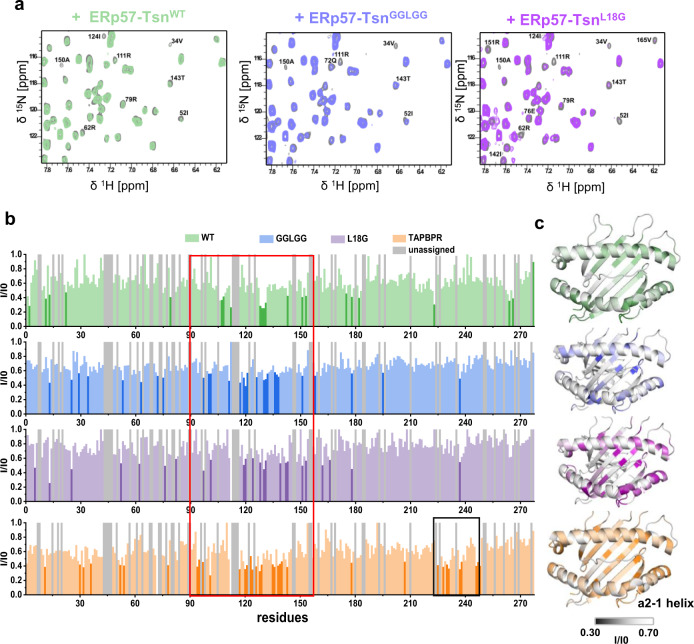
NMR analysis of tapasin mutants interacting with MHC-I. **a** Representative zoom view of the overlay of ^1^H-^15^N TROSY-HSQC spectra of 50 µM B*27:09/photoRL9 (gray) and in the presence of equimolar ERp57-Tsn^WT^ (green), ERp57-Tsn^GGLGG^ (blue) or ERp57-Tsn^L18G^ (purple). Attenuated peaks are marked. **b** Peak intensity ratio analysis (I/I0) of B*27:09/photoRL9 in the presence of ERp57-Tsn^WT^, ERp57-Tsn^L18G^, ERp57-Tsn^GGLGG^, and TAPBPR against B*27:09/photoRL9 (I0) are plotted against residue sequence. Significantly reduced residues (below I/I0 average – δ) were highlighted in corresponding dark color. Unassigned residues are plotted as gray bars. Red squared region means affected region in the peptide binding groove in the presence of tapasin or TAPBPR. Black squared region means region only affected by TAPPBR. **c** Peak intensity ratio (I/I0) are mapped as B-factor on the structure of B*27:09. The range of the intensity ratio is from 0.3 to 0.7 for all mutants. The color code is the same as in **b**. Unassigned residues are plotted as white color.

Next, in order to investigate if scoop loop mutants differently affect the interaction of tapasin with pMHC-I, we analyzed the affected residues of B*27:09/photoRL9 upon addition of ERp57-Tsn^GGLGG^ and ERp57-Tsn^L18G^ mutants by NMR spectroscopy. Similar to ERp57-Tsn^WT^, line broadening of a similar number of peaks were observed in the presence of both mutants. The binding groove residues significantly affected by ERp57-Tsn^GGLGG^ and ERp57-Tsn^L18G^ overlapped largely with residues affected by ERp57-Tsn^WT^ (Fig. 5b, the red boxed region).

Unexpectedly, residue T143 was also affected by adding ERp57-Tsn^L18G^ (Fig. 5a), which suggests that this mutation in the loop_11–__20_ does not significantly affect tapasin interacting with pMHC-I’s F-pocket region, although L18 is critical for the catalytic activity of tapasin. It is well known that T143 as well as Y84 form essential H-bonds with the carboxyl group of the bound peptide in the pMHC-I and we also observed these two H-bonds in the crystal structure of B*27:09/photoRL9 (Supplementary Fig. 6c). Thus, we hypothesize that the observed signal intensity reduction of T143 (Y84 is not assigned) is indicative of the destabilization of the H-bonds of the F-pocket region in the presence of tapasin.

As a comparison, we also analyzed the effect of TAPBPR on residues of B*27:09/photoRL9 by NMR spectroscopy. Similar to tapasin, no chemical shift changes but line broadening of peaks were observed. A stronger intensity reduction in the binding groove of MHC-I was detected in the presence of TAPBPR as compared to tapasin, especially for residues at the bottom of the F-pocket (Fig. 5b). In addition to residues in the loop_126–__133_, the N-terminus of the α_2-1_-helix was strongly affected by TAPBPR similar to what we observed for the UV-cleaved B*27:09/photoRL9 complex (Fig. 1d). In contrast to tapasin, peak intensity of residues in the β-sheet_225-232_ of the α3-domain of the MHC-I heavy chain were strongly reduced in the presence of TAPBPR (Fig. 5b, black boxed region), which is in line with the observed interaction between the IgG domain of TAPBPR and β-sheet_225-232_ of the α3-domain in the crystal structure of the MHC-I-TAPBPR complex^17,18^. This clearly suggests that the interaction between the IgG domain of TAPBPR with α3 domain of the heavy chain is stronger compared to tapasin, where we did not observe the corresponding signal loss (Fig. 5b).

## Discussion

**Fig. 6:**
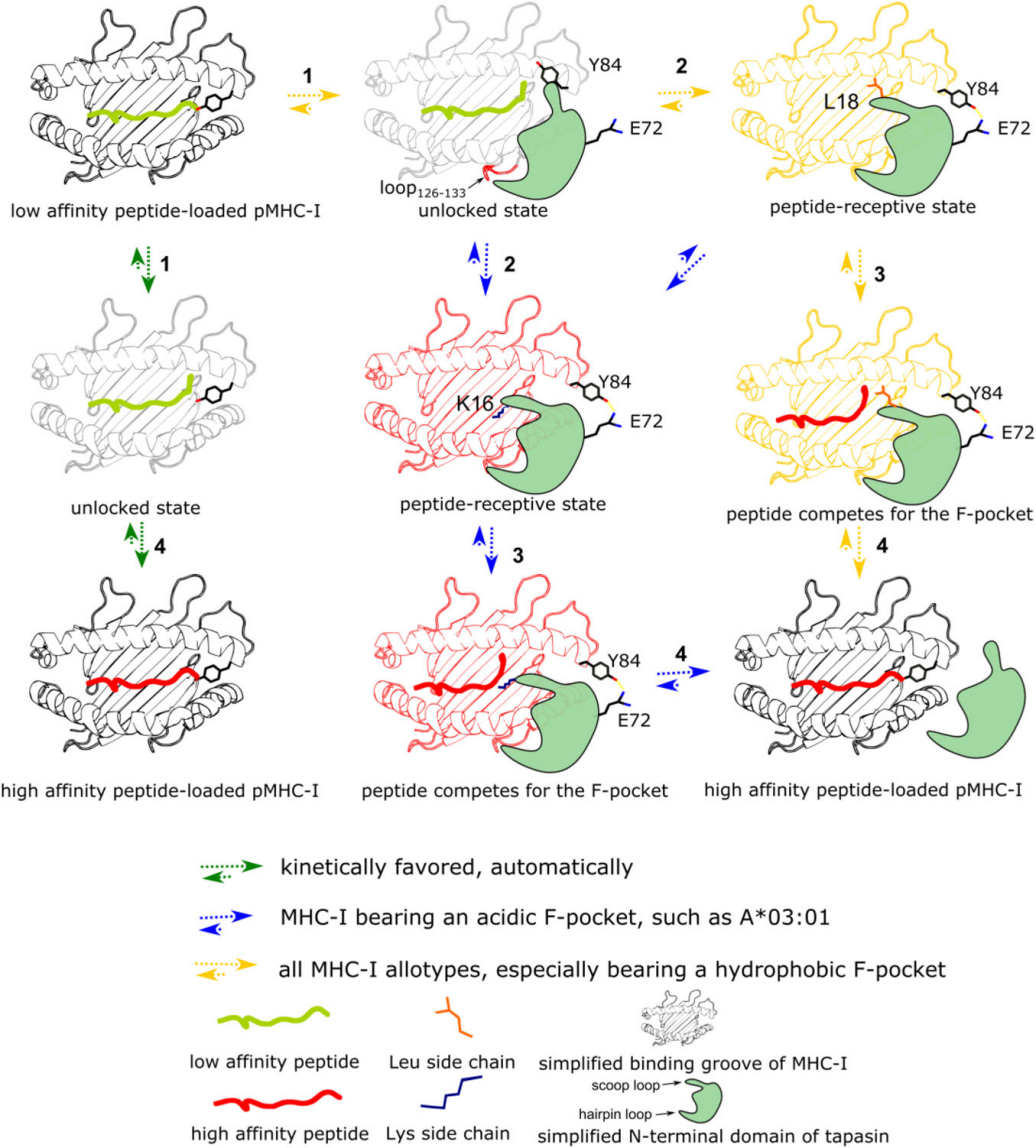
The proposed mechanism of tapasin-mediated peptide exchange. Step 1: pMHC-I molecules sample conformations, which determine peptide binding and tapapsin binding. Tapasin recognizes a certain state of pMHC-I by approaching pMHC-I from the α_2_-helix side of the pMHC-I. Interaction between its loop_187-196_ with the loop_126-133_ of heavy chain provides initial binding which alters the dynamics of the peptide binding groove and subsequently together with the interaction between its E72 in strands β3 and Y84 of the heavy chain promote the C-terminus of peptide to adopt loose binding in the F-pocket accompanied. Now pMHC-I switches to the unlocked state. MHC-I charged with very low affinity peptide can switch to peptide unlocked state automatically by the motion of the bound peptide adopting loose binding state. Step 2: The scoop loop_11-20_ interacts with the F-pocket region and stabilize the F-pocket with side chain of L18. Now pMHC-I switches to the peptide receptive state. For allotypes bearing a negatively charged F-pocket, tapasin can also use the residue K16 in the scoop loop_11-20_ to stabilize the charged F-pocket, which increases the efficiency of tapasin-mediated peptide exchange. Step 3: The upcoming peptide competes for the F-pocket until the affinity is high enough to replace the side chain. Step 4: Once the high-affinity peptide binds, tapasin is released from the pMHC-I.

Tapasin, a critical component of the PLC, acts as the peptide editor to shape the peptide pool presented by MHC-I on the cell surface. The cryo-EM structure of the PLC provides insight into how tapasin interacts with MHC-I as well as other PLC components. However, the molecular mechanism of tapasin-mediated peptide exchange of MHC-I remains elusive, given the inherent dynamics of critical regions within the complex. Here, we capitalize on NMR analysis to demonstrate that the dynamic properties of residues in the F-pocket are affected by tapasin binding and coincide with residues that underwent changes upon UV-induced peptide cleavage. A recent study reported that the switch between a peptide-free unlocked state and a peptide-bound locked state of the F-pocket is important for peptide binding^36^. Our results indicate that tapasin promotes peptide exchange of pMHC-I by switching the F-pocket to the unlocked state. T143, similar to Y84, forms a hydrogen bond to the terminal carboxylate group of the peptide and the observed line broadening of its NH group in the presence of tapasin (Fig. 1f) most likely reflects the loss of this H-bond as a consequence of partial peptide dissociation. Interestingly, in a recent MD simulation based on the entire PLC, E72 of tapasin was found to contact the conserved Y84^46–48^ and a E72K mutant shows impaired MHC-I binding compared to wild-type tapasin^16^. In the MHC-I-TAPBPR-structure, E105 which structurally corresponds to E72 in tapasin, is found to form an H-bond with Y84 of the MHC-I molecule, thereby replacing the H-bond formed with the C-terminus of the peptide^17,18^. Thus, we conclude that disruption of the C-terminal hydrogen bonds between the conserved residues Y84 and T143 of MHC-I and peptide represents a hallmark of tapasin action. Interestingly, we see NMR signal reduction of T143 in the presence of the catalytically inactive ERp57-Tsn^L18G^ mutant (Fig. 5). Thus, it must be concluded that the initial destabilization or even disruption of the hydrogen bonds between the MHC-I molecules and the C-terminus of the peptide are independent of the scoop loop. Unlocking of the F-pocket region conceivably allows the loop_11–__20_ of tapasin to place its L18 residue near or into the F-pocket (Fig. 6).

In addition to the altered dynamics of the F-pocket, the loop_126–__133_ of the heavy chain was also strongly affected by tapasin, as indicated by severe line broadening of residues in this region (Supplementary Fig. 2c). Interestingly, this loop_126–__133_ also shows conformational exchange in the CPMG experiments (Fig. 1b), indicating that a rare second conformation is already present in stable pMHC-I complexes. The loop_126-133_ region was observed to be contacted by the tapasin loop_187–__196_ in the PLC structure and mutations in both loops abrogate peptide editing in a cellular setting^15,16,41^, indicating the possible role of the interaction in forming the complex. Notably, both loops are also highly conserved among species (Supplementary Fig. 7a, b), implying that they represent an allotype-independent interface.

We investigated the function of human tapasin loop_11–__20_ in catalyzing peptide exchange of MHC-I in more detail and identified L18 as the critical residue in mediating peptide exchange, which is in line with published studies on the role of Leu both in the tapasin^21,37^ and TAPBPR scoop loop^20^. Three human allotypes with distinct F-pockets were studied to demonstrate the importance of L18 in all cases. In addition, we found that K16 contributes to the activity of tapasin for MHC-I allotypes bearing an acidic F-pocket. Our mutational studies indicate that the chemical nature as well as flexibility of the loop_11–__20_ tunes the catalytic activity of tapasin. The catalytic activity of ERp57-Tsn^L18G^ (SGKGG) was significantly lower than that of ERp57-Tsn^GGKGG^ towards A*03:01, although the only difference is a serine in position 14 of the tapasin loop in ERp57-Tsn^L18G^, indicating that the presence of a polar residue can also influence the allele-specific tapasin activity, at least for some alleles. We expected to see that the introduction of an additional Lys in position 18 would increase the activity of tapasin for A*03:01, which was not the case here. The reduced activity might be explained by the electrostatic repulsion between K16, K18, and K20 in the ERp57-Tsn^L18K^ loop.

As for the allelic differences in tapasin-catalyzed peptide exchange, we showed that the influence of tapasin on peptide association kinetics is positively correlated with the acidity of the F-pocket of the MHC-I, as is shown in detail with B*27:05 and A*03:01 F-pocket mutants (Supplementary Fig. 4d–h). Our results are consistent with the difference in tapasin dependence of the well-studied allotypes B*44:02 and B*44:05, which differ by only one residue at the bottom of the F-pocket: B*44:02, which harbors an Asp at position 116, is strongly tapasin dependent, while B*44:05, bearing a Tyr at position 116, is of low tapasin dependence. The positive correlation between the acidity of the F-pocket of MHC-I and tapasin enhancement on peptide exchange is also in agreement with results from a recently published paper in which highly tapasin-independent allotypes (e.g., A*68:01, A*02:02, A*02:01, B*81:01, B*35:01, and B*42:01) display hydrophobic F-pockets, while the tapasin-dependent allotypes (e.g., A*01:02, A*01:01, A*36:01, B*44:03, and B*44:02) contain an Asp at position 116^49^. For the less tapasin-dependent allotype A*02:01, increasing the F-pocket acidity did not enhance tapasin dependence (Supplementary Fig. 4j, k), suggesting that the scoop loop interaction is not the main determinant for catalytic activity in this case.

We explored the peptide exchange rates of B*27:09/05 with different photocleavable peptides for which affinities ranged from high to medium in the presence of Erp57-Tsn^WT^ (Supplementary Fig. 3). Our results indicate that the catalytic effect of tapasin is more pronounced when the affinity of the remaining 7-mer peptide is relatively high. In case the 7-mer peptide affinity is low, dissociation will be fast and not require tapasin. The conclusion that tapasin is more efficiently replacing medium-to-high-affinity peptides is in line with a report using a leucine zippered complex of HLA-B*08:01 and tapasin^6^ and a study that showed that tapasin catalyzes thermodynamically favored but kinetically disfavored exchange of suboptimal peptide for high-affinity peptides^40^.

Tapasin and TAPBPR utilize conserved structural features to allow for the initial steps of catalyzed peptide exchange. Conserved residues (E72 in tapasin corresponding to E105 in TAPBPR) in the catalysts provide an alternative hydrogen bonding pattern for F-pocket residue Y84, thereby facilitating release of the C-terminus of bound peptides. Interestingly, the dynamics of the F-pocket region were also affected by adding TAPBPR (Fig. 5b), suggesting TAPBPR and tapasin share similar mechanisms to turn MHC-I to a peptide-receptive state by modulating the dynamics in the F-pocket region. The loop_126–__133_ region also showed NMR line broadening in the presence of TAPBPR (Fig. 5b) and the loop_126-133_ was observed to form polar contacts with the “jack hairpin” loop of TAPBPR in complex with MHC-I^17,18^, implying that both molecules share a common mechanism where the dynamics of the loop_126-133_ of MHC-I molecules is sampled by the “jack hairpin” loop of either tapasin or TAPBPR. However, the affected region extends into the structurally proximal α_2-1_ helix in TAPBPR (Fig. 5b) which might reflect the 2 Å displacement of this helix seen upon complex formation^15,17,18^.

Several groups recently have investigated the function of the TAPBPR scoop loop^19–21^, and one of these studies found TAPBPR preferentially promotes peptide exchange of MHC-I allotypes bearing a hydrophobic F-pocket^28^. The authors proposed that L30 in the human TAPBPR loop is the key residue to mediate peptide dissociation^20^. Interestingly, the charge state of the mouse TAPBPR scoop loop is similar in sequence to human TAPBPR (Supplementary Fig. 8a) in line with the observed hydrophobicity of all mouse MHC-I alleles. In this respect it is interesting that a recent study indicates the long G24-R36 scoop loop of TAPBPR containing the critical leucine does not reach into the F-pocket but rather sits on top of it thereby acting as a kinetic trap for medium-to-high-affinity peptides under conditions where peptide concentrations are low^21^. Thus, while TAPBPR can enhance peptide exchange of high-affinity peptides during MHC complex maturation, it may also chaperone existing complexes along the transport through the ER and Golgi compartments when no high-affinity peptides are available.

Based on the finding that tapasin loop composition and flexibility is important for catalysis, we compared the tapasin scoop loop sequence of different species and found that L18 is conserved among species (Supplementary Fig. 8b). This agrees with our conclusion that L18 is the critical residue to mediate peptide exchange, which is also in line with a previous study showing that mutating Leu in the mouse tapasin loop_11–20_ significantly reduced the surface expression level of H2-K^b 34^. In contrast to the conserved Leu, there is no Lys in the mouse tapasin scoop loop, which coincides with none of the reported murine MHC-I allotypes to bear an acidic F-pocket. Furthermore, in our experiments the ERp57-Tsn^GGGGL^ mutant serves as a mimic of mouse tapasin and we find its activity to be higher for B27:09 compared to A*03:01 (Fig. 4b, c). Thus, according to our mechanistic findings, the emergence of a Lys residue in the human tapasin scoop loop is to enhance the catalytic activity of tapasin for human allotypes bearing an acidic F-pocket. Future research will have to show whether a possible co-evolution between tapasin and MHC-I allotypes has occurred in order to enhance the antigenic repertoire and immunological fitness of non-rodent mammalian species.

## Methods

### Refolding and purification of pMHC-I proteins

Human β_2_m (1-100), the luminal domain of HLA-A*02:01(1-276), HLA-A*03:01(1-274), HLA-B*27:05 (1-277), and HLA-B*27:09 (1-277) were expressed in BL21(DE3) *Escherichia coli* as inclusion body and purified as described below^50^: Cell pellets were resuspended with P1 lysis buffer (50 mM Tris, 1% (w/v) sodium deoxycholate, 1% (w/v) Triton X-100, 100 mM NaCl, 0.1% (w/v) NaN3, 0.5 mM MgCl_2_, 10 mM DTT, 20 µg/mL DNase I, pH 7.5) and sonicated for 30 min on ice water. The sonicated lysate was centrifuged at the speed of 12,000×*g* for 10 min and the supernatant was trashed. The white pellet is the inclusion bodies. Inclusion bodies were first washed twice with P2 buffer (50 mM Tris, 100 mM NaCl, 0.5% Triton X-100, 0.5 mM EDTA, 0.15 (w/v) NaN3, 10 mM DTT, pH 7.5) and one time with PBS buffer by sonicating for 10 min, followed by centrifuging at the speed of 12000 g for 10 min each time. In the last step, inclusion bodies were resuspended with P3 buffer (20 mM Tris, 0.5 mM EDTA, 4 M urea, 10 mM DTT, pH 8.0), dissolved overnight while rotating, centrifuged at the speed of 12000 g for 10 min to remove the unsolved particles. The supernatant is the desired denatured protein and was shock frozen and stored at −80 °C until use. Corresponding heavy chain mutants were generated by standard site directed mutagenesis using heavy chain WT as template, primers used were listed in Supplementary Table 4.

pMHC-I proteins were obtained by refolding heavy chain, β_2_m with high-affinity peptides at 4 °C as described below. Briefly: heavy chain, β2m and the corresponding restricted high-affinity peptide were refolded together in refolding buffer (0.4 M arginine, 0.1 M Tris, GSH:GSSG = 5 mM:0.5 mM, 2 mM EDTA, 0.5 mM PMSF, pH 8.0 for A*03:01 and A*02:01, pH 7.5 for B*27:05/09). For 1 L refolding, 10 mg peptide (photocleavable peptide photoKV9 for A*02:01 first need be dissolved in DMSO) was first added into pre-cooled refolding buffer, followed by dropwise adding 30 mg β2m. After stirring for 2 h at 4 °C, 30 mg heavy chain was dropwise added. The refolding bottle was then kept at 4 °C while stirring (for photocleavable peptides, the refolding bottle need be kept in dark) until purification. The refolding time varies for different allotypes: 3 days for A*02:01, 5 days for A*03:01, 7 days for B*27:09 (heavy chain equipped with or without a His-tag) and B*27:05 without His-tag, 14 days for B*27:05 when heavy chain bears a His-tag. Peptides used for refolding in this study were chemical synthetized (GL Biochem (shanghai) ltd. or DG peptides, China). Peptides’ sequence are also summarized in Supplementary Table 1: KILGFVFJ*V (photoKV9) for A*02:01, A*02:01Y116D, A*02:01H114D, A*03:01D116Y and A*03:01R114H/D116Y, KLIETYFJ*K (photoKK9) for A*03:01, A*03:01D116Y, A*03:01R114/HD116Y and A*02:01Y116D, RRKWRRWJ*L (photoRL9) for B*27:05/09, B*27:05D116Y and B*27:05H114R, IRAAPPPJ*F (photoIF9) for B*27:05/09, where J* = 3-amino-3-(2-nitrophenyl)-propionic acid^50^, RRKWRRWHL (RL9) for B*27:05/09. Refolded pMHC-I complexes were purified by size-exclusion chromatography (SEC) with a Superdex 200 increase 10/300 GL column in PBS buffer. Pure fractions were pooled, shock frozen and stored at −80 °C until use. The T_m_ of pMHC-I molecules (WT) before and after UV exposure are summarized in Supplementary Table 2.

### Expression and purification of ERp57-Tsn mutants and TAPBPR

The construct expressing ERp57-Tsn^WT^ was similar as previously described^16^. Briefly, tapasin (1-398) fused with a C-terminal 6xHis-tag was inserted into pFastBac-Dual vector under the polyhedrin promoter between *BamH*I/*Xba*l and ERp57 (1-504, C60A) was inserted under the p10 promotor between *Sma*I/*Sph*I. All scoop loop mutants of ERp57-Tsn were generated by standard site directed mutagenesis using ERp57-Tsn^WT^ as template (unless otherwise stated). Primers used were listed in Supplementary Table 4. TAPBPR (1-405) fused with a C-terminal 6xHis-tag was inserted into pFastBac1 vector between *BamH*I and *Xba*l, C97A mutation was introduced to increase the expression yield of TAPBPR while didn’t affect the activity of TAPBPR^12^. Constructs expressing ERp57-Tsn^WT^, its mutants and TABPPR were transfected into Sf9 (*Spodoptera frugiperda*) insect cells based on the manufacturer’s protocol (Bac-to-Bac® Baculovirus Expression System, Thermo Fisher) to generate virus stock. Expression of ERp57-Tsn proteins were verified by dot plot with anti-His-tag antibody (anti-His-HRP, dilution 1:2000, Miltenyi Biotec, Mat. No.: 120-003-811, Lot No.:5170502017).

Insect cells were harvested 4 days after transfection and cell pellets were lysed in cold lysis buffer (100 mM KH_2_PO_4_, 600 mM KCl, 1% Triton X-100, pH = 8.0). Protease inhibitor, DNase I, MgCl_2_ and 5 mM MMTS (MMTS: S-methyl methanethiosulfonate, Sigma-Aldrich, Switzerland, not add when purifying TAPBPR) were freshly added. Supernatant was purified with Protino® Ni-NTA Agarose (Macherey-Nagel™, Germany) by spin column purification method. Briefly, the lysate supernatant (20 ml) was incubated with 0.4 ml buffer equilibrated nickel beads at 4 °C for 30 min–1 h (Ni-NTA Agarose, Macherey-Nagel, Germany) while rotating. Nickel beads were spun down at the speed of 500×*g* for 5 min and then transferred into a 2-ml eppendorf tube. Nickel beads were washed with 1 ml equilibrium buffer (100 mM KH_2_PO_4_, 600 mM KCl, pH = 8.0) three times and then with 1 ml wash buffer (10 mM imidazole, 100 mM KH_2_PO_4_, 600 mM KCl, pH = 8.0) each time until the A280 absorption lower than 0.2. Protein was eluted with 0.4 ml elution buffer (150 mM imidazole, 100 mM KH_2_PO_4_, 600 mM KCl, pH = 8.0) each time until the A280 absorption below 0.1. Eluted fractions were pooled and further purified by Superdex 200 increase 10/300 GL column in PBS buffer. Purified tapasin mutants and TAPBPR were analyzed by non-reducing 4–12% SDS-PAGE (Supplementary Fig. 5d, also see source data file). Pure fractions were pooled, shock frozen and kept at −80 °C until use.

### Thermoshift measurements

Sypro-orange dye was used to determine the T_m_ of proteins. In all, 20 μl protein (0.2–0.4 mg/ml) was mixed with 5 μl Sypro-orange dilution buffer (243.5 μl buffer + 1.5 μl Sypro-orange, Invitrogen, USA), then added to the MicroAmpROptical 96-well Reaction Plate (Life Technologies), measured on the qPCR machine (Stratagene Mx3005PTM, ThemoFisher) using a protocol with heating at the speed of 2 °C/min from 25 °C to 95 °C. Each sample was measured in three replicates. Experimental data analysis was processed with Origin 2019 and T_m_ was obtained by fitting with the Boltzmann function.

### NMR chemical shift assignments

All NMR spectra (unless otherwise stated) were acquired on a Bruker Avance III 700 MHz spectrometer equipped with a 5-mm triple resonance cryoprobe. Spectra were processed with TopSpin3.2 (Bruker, Billerica, USA) and analyzed with CcpNmr Analysis 2.4.2^51^. All NMR samples were measured in PBS 7.2 buffer with 10% D_2_O at 300 K (unless otherwise stated).

The sequence of B*27:09 heavy chain for preparing NMR samples was the same as the BMRB Entry 25713. In order to confirm backbone amide assignments of the B*27:09 heavy chain, B*27:09 heavy chain was isotope-labeled with ^15^N, ^13^C, and ^2^H. For 1 L deuterated M9 medium culture, 1 g ^15^NH_4_Cl and 1.5 g d-glucose-U-^13^C6,1,2,3,4,5,6,6-d7 (Cambridge Isotope Laboratories, Inc.) were used. The inclusion body was then purified in protonated buffer to allow for ^1^H-^15^N-detection. The backbone amide assignments of B*27:09 heavy chain were transferred from available assignments for B*27:09/RL9 (BMRB Entry 25713) and confirmed with HNCA, HNCO, and ^1^H-^15^N-TROSY-HSQC spectra with ^15^N-^13^C-^2^H B*27:09/RL9 (270 μM). ^1^H-^15^N-TROSY-HSQC spectrum of ^2^H-^15^N B*27:09/photoRL9 was recorded at a concentration of 250 μM and the assignments were transferred from the confirmed assignments of ^15^N-^13^C-^2^H B*27:09/RL9 (Supplementary Fig. 9).

In order to investigate methyl-group dynamics of B*27:09, Ile, Val, and Leu methyl groups were ^1^H-^13^C labeled in a deuterated ^15^N/^12^C background. For 1 L deuterated M9 medium culture, 100 mg precursor for Val and Leu (2-keto-(3-methyl-^13^C)-butyric-4-^13^C,3-d acid sodium salt, Santa Cruz Biotechnology, Inc) and 50 mg precursor for Ile (2-ketobutyric acid-4−^13^C,3,3-d2 sodium salt hydrate, Sigma-Aldrich, USA), 1.5 g ^15^NH_4_Cl and 1.5 g d-glucose-U-^12^C6,1,2,3,4,5,6,6-d7 were added. ILV methyl assignments were obtained with a {I(^13^CH_3-_ only), L(^13^CH_3_,^12^CD_3_), V(^13^CH_3_,^12^CD_3_)} U-[^15^N,^13^C,^2^H] sample of B*27:09/RL9 at 310 K, using a HMCM[CG]CBCA spectrum^52^ acquired at a Bruker AV-III 600 spectrometer using a TCI-cryoprobe equipped with one-axis self-shielding gradients. The sample had a concentration of 26 mg/ml in 10 mM phosphate pH 7.5, 150 mM NaCl, and 10% D_2_O and methyl assignments are updated in BMRB entry 25713. These assignments as well as those available for A*02:01 (BMRB Entry 27631) and A*01:01 (BMRB Entry 27632) were used to transfer assignments to other complexes used in this publication^30^. Transferred assignments were validated where possible by methyl-methyl and methyl-amide NOEs with 170 µm ^1^H-^13^C-ILV-^15^N-^2^H B*27:05/photoRL9 in combination with the crystal structure of B*27:05/RL9 (PDB ID 1OGT). The ^1^H-^13^C HSQC spectra of B*27:09/RL9 and B*27:09/photoRL9 are shown in Supplementary Fig. 9.

### CPMG relaxation dispersion

Carr-Purcell-Meiboom-Gill (CPMG) relaxation dispersion experiments of 150 μM ~ 250 μM ^1^H-^13^C-ILV-^15^N-^2^H B*27:09/photoRL9 were recorded single-scan interleaved using an INEPT transfer for excitation^53^ and a WATERGATRE element for water suppression in order to measure in 10% D_2_O. CPMG pulse frequencies of 0, 950, 50, 300, 150, 800, 100, 250, 400, 600, 200, 50, 500, 300, and 700 with a constant time delay of 40 ms were used. Peak intensities were converted to the ${R}_{2}^{{{\mathrm{eff}}}}$ transverse decay rates with the equation${R}_{2}^{{{\mathrm{eff}}}}=1/{T}_{{{\mathrm{CPMG}}}}\times {\rm{ln}}({I}_{0}/{I}_{{{\mathrm{CPMG}}}})$. Only assigned peaks were analyzed. CPMG profiles of all methyl groups displaying dispersion (${R}_{2}^{{{\mathrm{eff}}}}$ > 2 s^-1^) were fitted to a two-state model using the program NESSY^54^.

### HSQC spectra after UV exposure and in the presence of ERp57-Tsn

^1^H-^15^N-TROSY-HSQC spectra of 50 µM ^1^H-^13^C-ILV-^15^N-^2^H B*27:09/photoRL9 were recorded before and after UV exposure. Peak intensity ratios (I/I0) were calculated from spectra before (I0) and after (I) UV exposure.

In order to study how B*27:09/photoRL9 would be affected by tapasin in solution, ^1^H-^15^N-TROSY-HSQC spectra of 50 μM ^1^H-^13^C-ILV-^15^N-^2^H B*27:09/photoRL9 in the absence (I0) and presence (I) of 50 μM un-labeled ERp57-Tsn^WT^ purified in matched buffer (PBS 7.2) were recorded and peak intensity ratios (I/I0) were calculated.

### Fluorescence Polarization assay

Peptide exchange assays of MHC-I were done with UV-exposed MHC-I refolded with photocleavable peptide (photo-MHC-I) exchanging against FITC-labeled high-affinity peptides in the absence or presence of ERp57-Tsn^WT^, its mutants or TAPBPR. All proteins used were purified in the same buffer (PBS pH 7.4, unless otherwise stated). Briefly: 20 μl of a 1 μM purified photo-MHC-I was UV exposed (arc-UV lamp, Newport 6295NS, filter 345 nm, 1000 W) on ice for 5 min at a distance of 15 cm and then added to the 384-well assay plate (Corning® 384-well Low Flange Black Flat Bottom Polystyrene NBS Microplate 3575, USA). In total, 10 μl of a 2 μM ERp57-Tsn^WT^ or mutants or 10 μl PBS buffer were added and then 10 μl of a 40 nM corresponding FITC-peptide were added right before measurement. Wells containing only FITC-peptide (final 10 nM) were measured as baseline. Each well has a total volume of 40 μl and experiment was performed at room temperature with three replicates. FP signals were recorded on PerkinElmer VICTOR^3^V (Fig. 4b, Supplementary Fig. 1a, and Supplementary Fig. 2) or recorded on Tecan Spark plate reader (Figs. 2b and 3b and Supplementary Fig. 4) with an excitation filter of *λ*_ex_ =  485 nm and an emission filter of λ_em_ = 535 nm and monitored over time range from 1 to 5 h. FITC-labeled high-affinity peptides used in this study are GILG-Lys^FITC^VFTV for A*02:01, KILG-Lys^FITC^VFTV for A*02:01, A*02:01Y116D, KLIE-Lys^FITC^YFSK for A*03:01, KLIE-Lys^FITC^YFSV for A*03:01D116Y, A*03:01R114HD116Y, A*02:01, and A*02:01Y116D, RRKW-Lys^FITC^RWHL for B*27:05/09, B*27:05D116Y and B*27:05H114R, and IRAA-Lys^FITC^PPLF for B*27:05/09 (summarized in Supplementary Table 1). Experimental data analysis was processed with Origin 2019 and the observed association rate *K*_*on*_ was calculated by using fitting function $Y={Y}_{0}-A{e}^{-{Kt}}$, where *Y*_*0*_ is the maximum value and A is the increased FP range.

### X-ray crystallography

Sample for crystallography was purified two times by SEC in 20 mM Tris/HCl pH 7.4, 150 mM NaCl, 0.01% sodium azide, and concentrated to 13–15 mg/ml as measured by the absorbance at 280 nm. Crystals were obtained by the sitting-drop vapor-diffusion method at 18 °C with a reservoir solution composed of 20% (w/v) polyethylene glycol 3350, and 200 mM KSCN. Crystal growth and diffraction quality was improved by microseeding. Prior to flash cooling of the crystals in liquid nitrogen, glycerol was added to the mother liquor to a final concentration of 25% (w/v).

Synchrotron diffraction data were collected at the beamline 14.1 of the MX Joint Berlin laboratory at BESSY II (Berlin, Germany). X-ray data collection was performed at 100 K. Diffraction data were indexed and processed with the XDS. Data collection and refinement statistics are given in supplementary Table 1. The phase problem was solved by molecular replacement using the program PHASER^55^ with the structure of HLA-B*27:09/pVIPR as search model (PDB ID 5IB5^56^). The structure was initially refined by applying a simulated annealing protocol and in later refinement cycles by maximum-likelihood restrained refinement using in PHENIX^57,58^. Model building and water picking was performed with COOT^59^. Intermediate and final structures were evaluated with and MOLPROBITY^60^ and the JCSG validation server (JCSG Quality Control Check v3.1). Figures were prepared with PyMOL (Version 1.8 Schrödinger, LLC). Atomic coordinates and structure factor amplitudes have been deposited in the Protein Data Bank under accession code 7ALO.

### Mass-spectrometric analysis

UV-exposed photo-MHC-I was purified by SEC to remove any possible fragment. In order to detect fragments of the photocleavable peptide, SEC fractions were analyzed by matrix-assisted laser desorption ionization-time of flight mass spectrometry (MALDI-TOF-MS) using α-cyano-4-hydroxycinnamic acid (HCCA) as matrix. The samples were applied on the MALDI target using the dried-droplet technique. Spectra were recorded on an Ultraflex-II TOF/TOF instrument (Bruker Daltonics, Bremen, Germany) in the positive reflector mode in the *m*/*z* range of 600–4000. Samples of photocleavable peptide before and after UV exposure served as a reference.

### Modeling of the tapasin scoop loop

The EM structure of the PLC, PDB ID 6ENY) served as a scaffold to build the MHC-I-ERp57-Tsn models using the crystal structures of MHC- I allotypes HLA-A*02:01 (PDB ID 1OGA), HLA-A*03:01 (PDB ID 2XPG), HLA-B*27:05 (PDB ID 1OGT), HLA-B*27:09 (PDB ID 1OF2), and the ERp57-Tsn complex (PDB ID 3F8U). The unresolved atoms in the tapasin scoop loop were modeled and refined with the MODELLER software^61,62^ using the corresponding sequence to generate an ensemble of 100 models for each allotype. With the Amber 12 simulation package, each MHC-I-ERp57-Tsn model was placed in an octahedral TiP3^63–65^ water box and neutralized with counterions. Then, each complex was energy minimized, positionally restrained (25 kcal mol^−1^Å^−2^), and heated from 100 to 300 K. The restraints were resolved in five steps, and then each complex was equilibrated for 2 ns. Short-range nonbonded interactions were taken into account up to a cut-off value of 9 Å.

### Modeling and scoring of tapasin mutants

The structural modeling of the tapasin mutants was performed with PyRosetta using the “ref2015” energy function^43–45^. The desired scoop loop sequence was computationally introduced into tapasin, followed by Monte Carlo-based simulated annealing sidechain and backbone minimization steps. The energy scores were calculated with the “score_jd2” application and averaged for all generated models per mutant.

### Reporting summary

Further information on research design is available in the Nature Research Reporting Summary linked to this article.

## Supplementary information

Supplementary Information

Reporting Summary

## Data Availability

NMR assignments for methyl groups of ILV of 27:09 heavy chain have been updated into the Biological Magnetic Resonance Data Bank (http://www.bmrb.wisc.edu) under accession number 25713. The X-ray structure of B*27:09 in complex with photoRL9 is deposited in the RCSB Protein Data Bank with accession codes PDB ID 7ALO [https://www.ebi.ac.uk/pdbe/entry/pdb/7alo]. Previously solved structures used in our study have the accession codes: 1OGT [https://www.ebi.ac.uk/pdbe/entry/pdb/1ogt], 1OF2 [https://www.ebi.ac.uk/pdbe/entry/pdb/1of2], 5IB5 [https://www.ebi.ac.uk/pdbe/entry/pdb/5ibu] 3F8U [https://www.ebi.ac.uk/pdbe/entry/pdb/3f8u) 6ENY [https://www.ebi.ac.uk/pdbe/entry/pdb/6eny]. Peptides used for motif generation were downloaded from IEDB (https://www.iedb.org/). MHC-I sequences used for analysis were download from https://www.ebi.ac.uk/ipd/imgt/hla/. Sequences of tapasin and TAPBPR from different species were downloaded from Uniprot (https://www.uniprot.org/). Other data are available from the corresponding author upon reasonable request. Source data are provided with this paper.

## Source data

Source Data
