# Supplementary material for: Exchange catalysis by tapasin exploits conserved and allele-specific features of MHC-I molecules
Source: Nat Commun. 2021 Jul 9;12:4236. doi: 10.1038/s41467-021-24401-4 (PMC8271027; doi:10.1038/s41467-021-24401-4)
Supplement: Supplementary file 1 — Supplementary Information [file 41467_2021_24401_MOESM1_ESM.pdf]

Exchange catalysis by tapasin exploits conserved and allele-specific features of MHC-I molecules

Huan Lan[1], Esam T. Abualrous[1,5], Jana Sticht[1,2], Laura Maria Arroyo Fernandez[1], Tamina Werk[1], Christoph Weise[1,2], Martin Ballaschk[3], Peter Schmieder[3], Bernhard Loll[4] & Christian Freund[1]*

[1]Laboratory of Protein Biochemistry, Institute for Chemistry & Biochemistry, Freie Universität Berlin, Thielallee 63, 14195 Berlin, Germany.

[2]Core Facility BioSupraMol, Institute for Chemistry & Biochemistry, Freie Universität Berlin, Takustr. 3, 14195 Berlin, Germany.

[3]Leibniz-Forschungsinstitut für Molekulare Pharmakologie, Robert-Roessle-Str. 10, 13125 Berlin, Germany.

[4]Laboratory of Structural Biology, Institute for Chemistry & Biochemistry, Freie Universität Berlin, Takustr. 6, 14195 Berlin, Germany.

[5]Artificial Intelligence for the Sciences, Department of Mathematics and Computer Science, Freie Universität Berlin, Arnimallee 14, 14195 Berlin, Germany.

Correspondence to: christian.freund@fu-berlin.de

Supplementary Figure 1

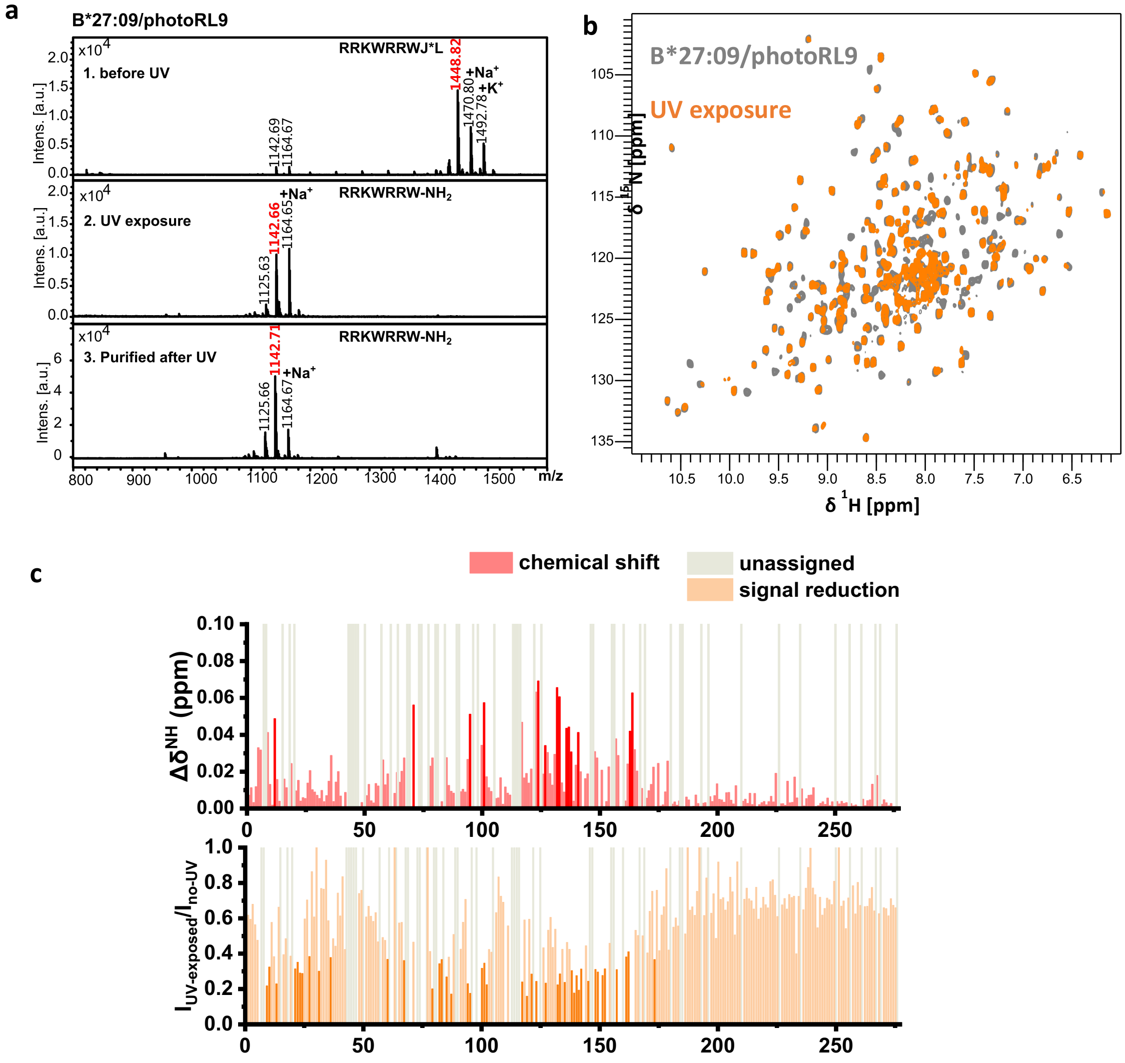

Supplementary Figure 1. Dynamic changes of B*27:09/photoRL9 after UV exposure. a) The average relative abundance for the selected mass range of MALDI-TOF-MS analysis of B*27:09/photoRL9 before UV-exposure, after UV-exposure and purified after UV-exposure. 7-mer fragment peptide was still detected in the UV-exposed MHC-I after gel-filtration chromatography purification. The signal of intact peptide and 7-mer fragment RRKWRRW-NH_2 (observed mass 1,142.66 Da, expected mass 1,143.47 Da) is noted in red as well as $+Na^+$ and $+K^+$ signals. **b)** Overlay of ^1H-^{15}N TROSY-HSQC spectra of B*27:09/photoRL9 (grey) and UV-exposed B*27:09/photoRL9 (orange). **c)** Chemical shift deviations (CSD, $\Delta\delta^{NH}$, p.p.m.) (top) and intensity changes ($I_{UV\text{-}exposed}$ /$I_{no\text{-}UV}$) (bottom) for peaks of UV-exposed B*27:09/photoRL9 compared to B*27:09/photoRL9. Residues showing significant CSD (1σ higher than the average CSD) are labelled in red. Residues showing significant intensity reduced signals (1σ less than the average intensity) are labelled in orange. Unassigned residues are plotted as grey bars. Raw data see source data file.

Supplementary Figure 2

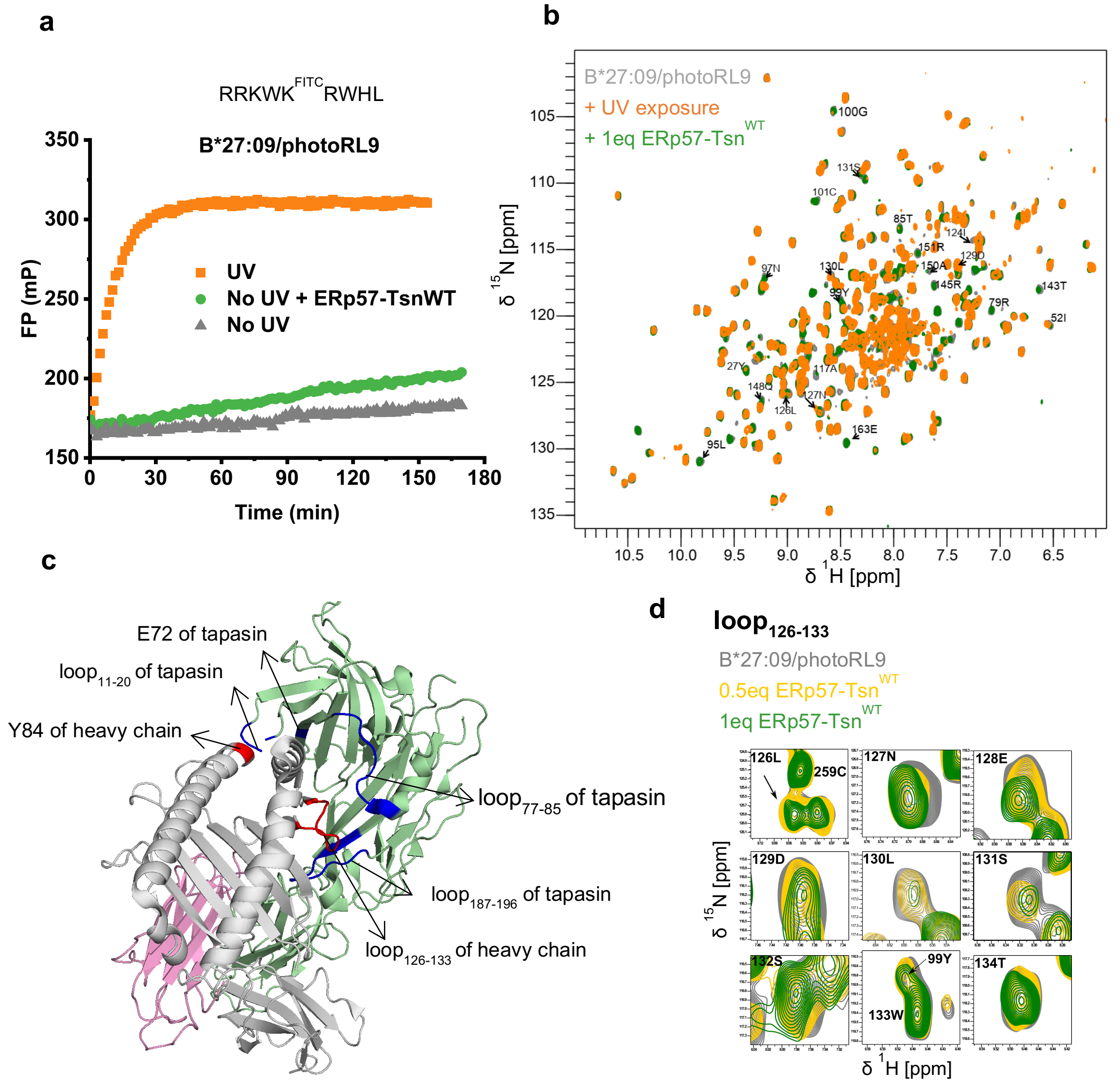

Supplementary Figure 2. NMR analysis of interactions between ERp57-Tsn and pMHC-I. a) Representative peptide exchange profiles of 500 nM 27:09/photoRL9 without UV-exposure (grey), in the presence of equimolar ERp57-Tsn^{WT} (green) and after UV-exposure (orange). **b)** Overlay of ^{1}H-^{15}N TROSY-HSQC spectra of B*27:09/photoRL9 (grey), in the presence of equimolar ERp57-Tsn^{WT} (forest) and UV-exposed B*27:09/photoRL9 (orange). Signal attenuated residues are marked. **c)** Structural elements that are important for the interaction between tapasin and MHC-I are colored in red for the heavy chain and in blue for tapasin. Structure of the MHC-I-tapasin complex is based on the structure of the PLC (6ENY). The heavy chain is displayed in grey, tapasin in green and β2m in pink. **d)** Representative peaks of residues in the loop$_{126-133}$ showing reduced signal in the presence of ERp57-Tsn^{WT} (0.5eq in yellow, 1eq in forest).

Supplementary Figure 3

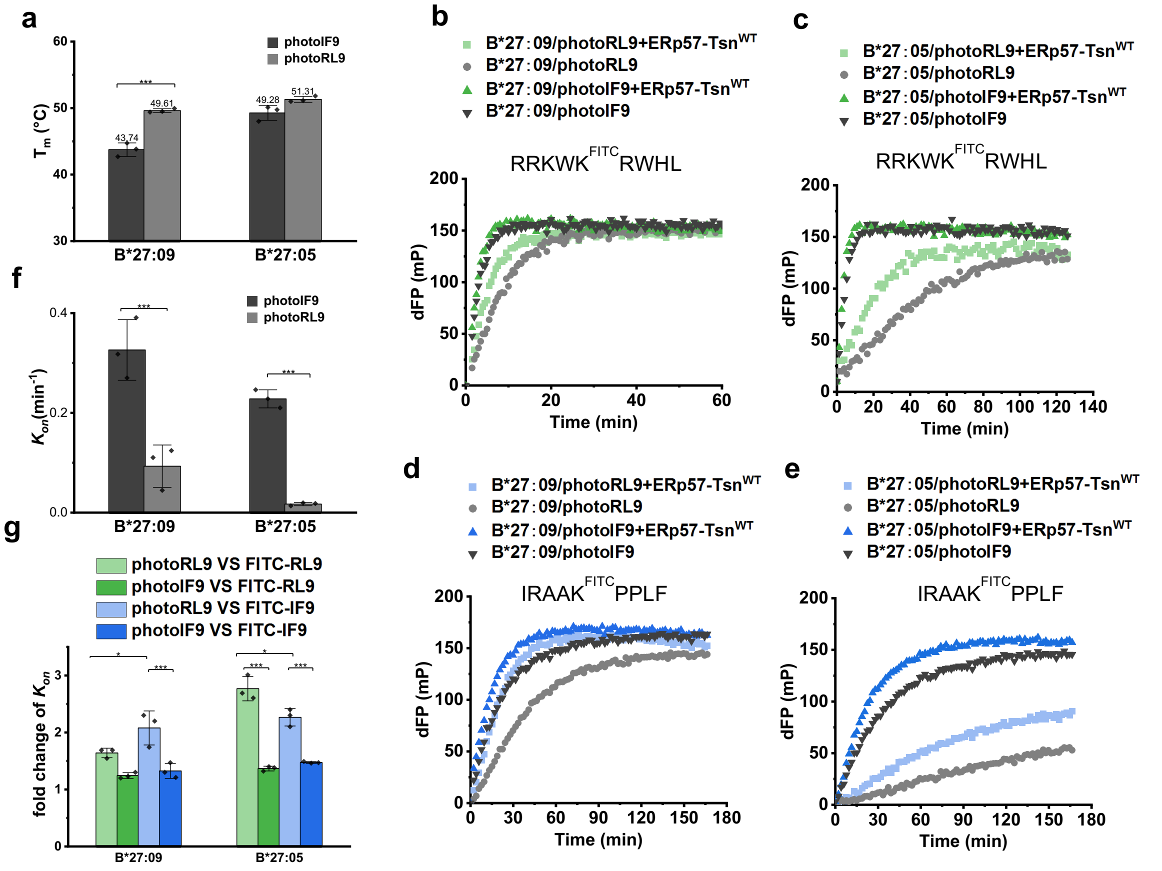

Supplementary Figure 3. Influence of tapasin on peptide exchange kinetics of MHC-I. a) The T_m values of B*27:09/05 in complex with photoIF9 (dark) or photoRL9 (grey) after UV-exposure. **b-e)** Peptide exchange profile of 500 nM B*27:09/photoRL9 (grey) and B*27:09/photoIF9 (dark) in the absence or presence of equimolar ERp57-Tsn after UV-exposure against FITC-RL9 (**b**) or against FITC-IF9 (**d**). Peptide exchange profile of 500 nM B*27:05/photoRL9 (grey) and B*27:05/photoIF9 (dark) in the absence or presence of equimolar ERp57-Tsn after UV-exposure against FITC-RL9 (**c**) or against FITC-IF9 (**e**). dFP means the baseline was subtracted. **f)** The uncatalyzed K_{on} of B*27:09/05 in complex with photoIF9 (dark) and photoRL9 (grey) exchange against FITC-RL9 after UV-exposure. **g)** Fold changes of K_{on} of B*27:09/05 in complex with photoRL9 and photoIF9 against FITC-RL9 or FITC-IF9 in the presence of tapasin. In **b-e)** FP assays were measured at the pH = 7.0. In **a) f) g),** errors bars represent SD and were calculated form three repeats, for comparison, the two-sample unequal variance Student's t-test was performed, *$p < 0.05$, **$p < 0.01$, ***$p < 0.001$.

Supplementary Figure 4

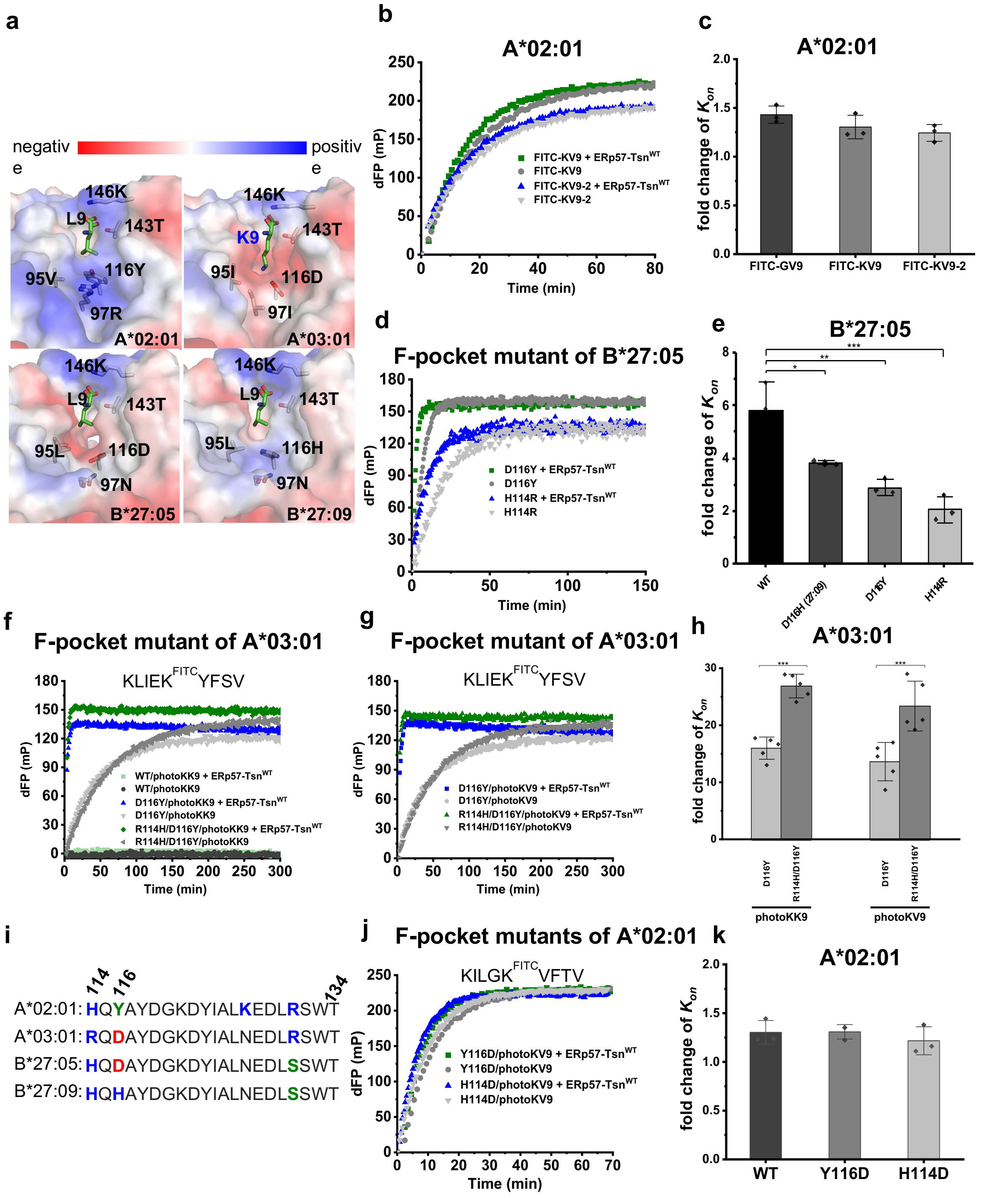

Supplementary Figure 4. The influence of tapasin on MHC-I peptide association kinetics is positively correlated with the acidity of its F-pocket. a) The F-pocket region of allotypes used in this study. The F-pockets of A*02:01 (PDB ID 1OGA), A*03:01 (PDB ID 3RL1), B*27:05 (PDB ID 1OGT) and B*27:09 (PDB ID 1OF2) are shown in surface representation colored by their vacuum electrostatics, highlighting the critical amino acids that compose the F-pocket as well as the peptide C-terminus residue (green) diving into the pocket in respective crystal structures. **b)** Peptide exchange profiles of UV-exposed A*02:01/photoKV9 against different FITC-peptides in the absence and presence of ERp57-Tsn^{WT}. FITC-KV9 is KILGK^{FITC}VFTV, FITC-KV9-2 is KLIEK^{FITC}YFSV. **c)** Fold changes of K_{on} of A*02:01/photoKV9 enhanced by ERp57-Tsn^{WT} against FITC-GV9, FITC-KV9 and FITC-KV9-2. FITC-

GV9 is GILGK^{FITC}VFTV. **d)** Peptide exchange profiles of F-pocket mutants B*27:05D116Y/photoRL9 and B*27:05H114R/photoRL9 exchange against FITC-RL9 in the absence and presence of ERp57-Tsn^{WT}. **e)** Fold changes of K_{on} of F-pocket mutants of B*27:05 enhanced by ERp57-Tsn^{WT}. **f)** Peptide exchange profiles of A*03:01 and its F-pocket mutants A*03:01D116Y and A*03:01R114H/D116Y refolded with photoKK9 exchange against FITC-KV9-2. **g)** Peptide exchange profiles of A*03:01D116Y and A*03:01R114H/D116Y refolded with photoKV9 exchange against FITC-KV9-2. **h)** Fold changes of F-pocket mutants of A*03:01 enhanced by ERp57-Tsn^{WT}. **i)** Sequence (residues 114-134) alignment of four allele used in this study**. j)** Peptide exchange profiles of A*02:01 F-pocket mutants refolded with photoKV9 against FITC-KV9 in the presence or absence of ERp57-Tsn^{WT}. **j)** Fold changes of K_{on} of A*02:01 mutants enhanced by ERp57-Tsn^{WT}. In **c) e) k)** Error bars (SD) were calculated from 3 independent experiments, in **h)** Error bars (SD) were calculated from 5 independent experiments, in **c) e) k) h)** for comparison, the two-sample unequal variance Student's t-test was performed, *p < 0.05, **p < 0.01, ***p < 0.001.

Supplementary Figure 5

a

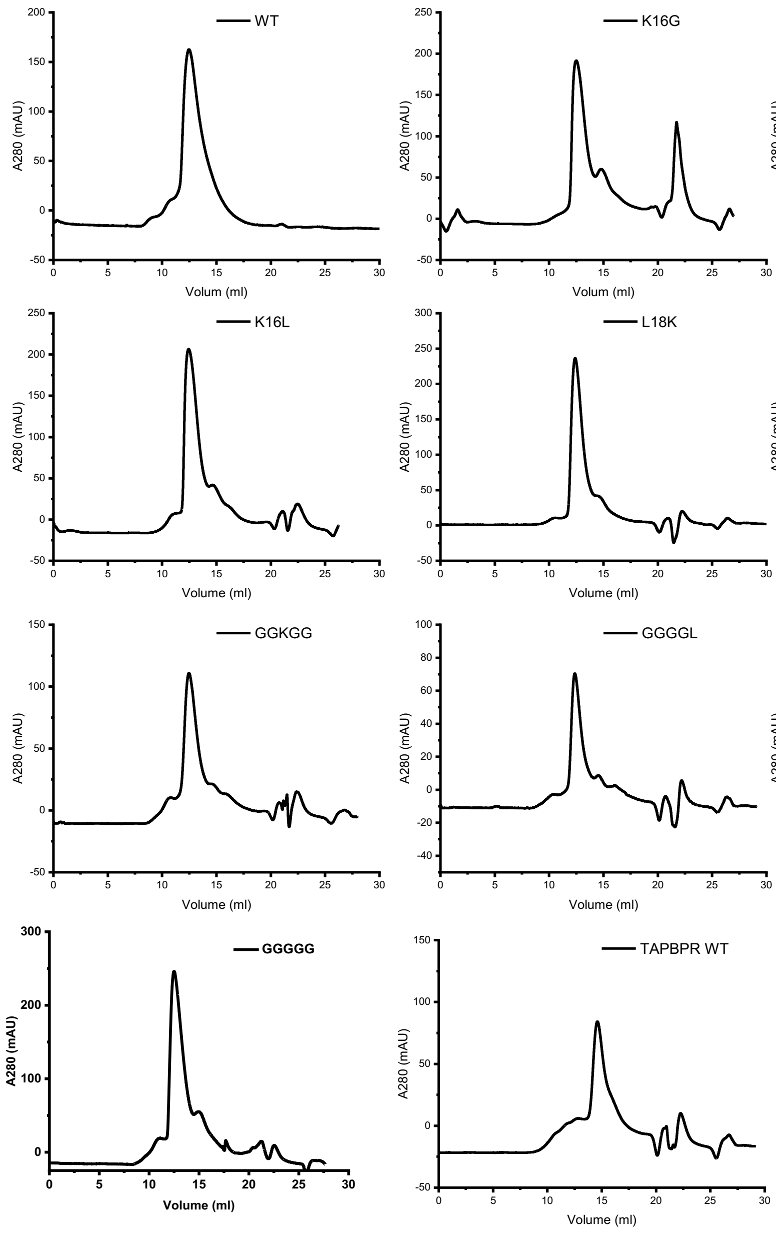

b

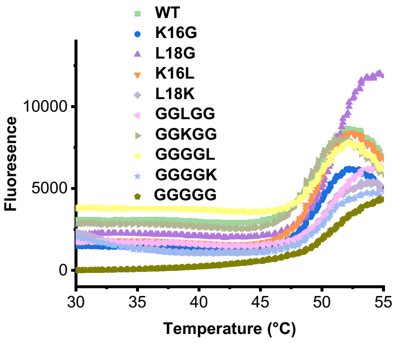

c

mutants	T_m (°C)
WT	49.33 ± 0.02
K16G	49.60 ± 0.03
L18G	50.85 ± 0.01
K16L	49.64 ± 0.02
L18K	49.36 ± 0.03
GGLGG	50.80 ± 0.02
GGKGG	48.85 ± 0.03
GGGGL	49.70 ± 0.03
GGGGK	49.52 ± 0.03
GGGGG	49.75 ± 0.05
TAPBPR	52.69 ± 0.03

d

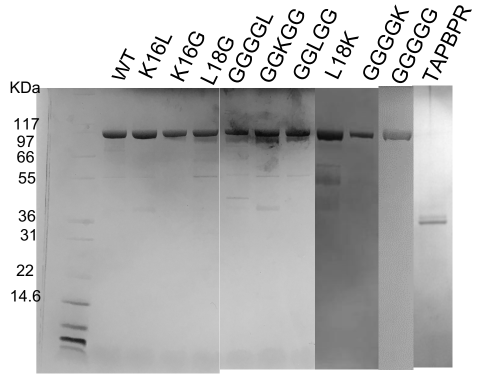

Supplementary Figure 5. Characterization of Tapasin mutants and TAPBPR. a) Chromatography profiles of ERp57-Tsn mutants and TAPBPR run in Superdex200 increase 10/300 GL column. **b)** The melting curves of tapasin mutants. **c)** The melting temperature T_m values of tapasin mutants. **d)** Non-reducing SDS-PAGE analysis of purified tapasin mutants and TAPBPR. Marker12^{TM} was used as marker. Uncropped gels see in Source Data file.

Supplementary Figure 6

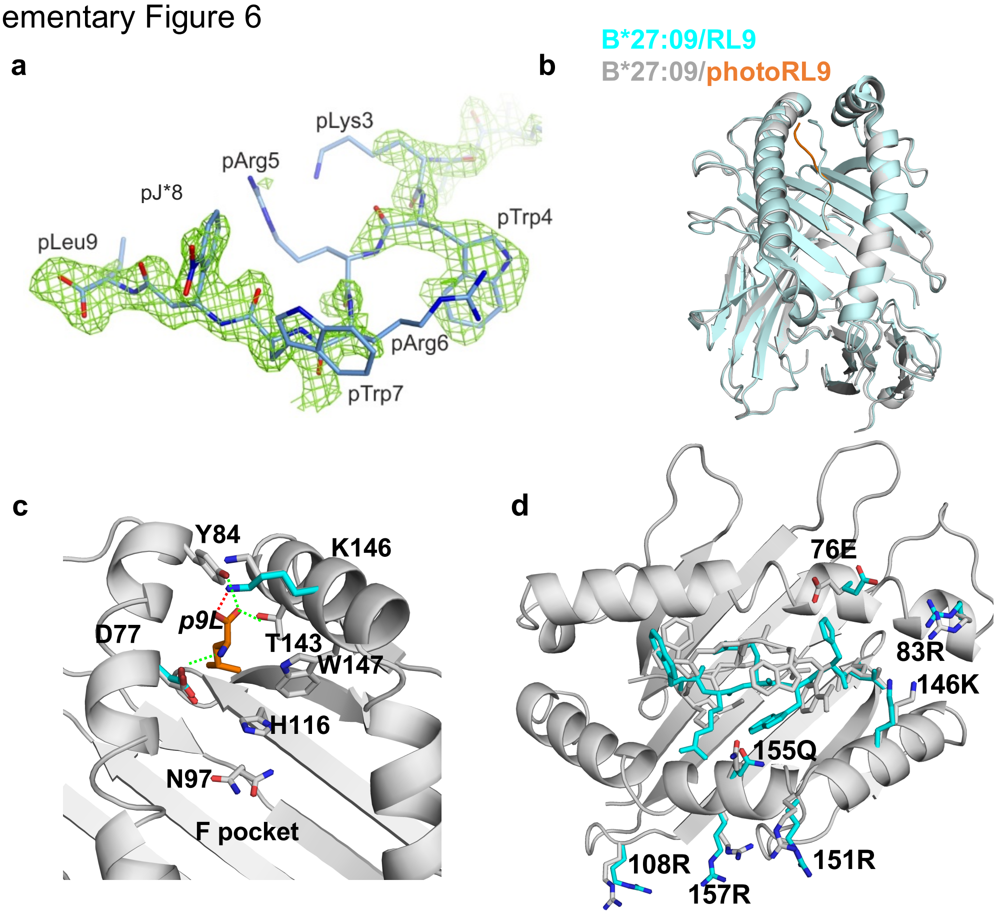

Supplementary Figure 6. Characterization of B* 27:09/photoRL9 by X-ray. a) stereo view of part of the electron density map. A POLDER map[65] was calculated for the omitted peptide. The electron density map is shown as green mesh contoured at 3 σ**. b)** Crystal structure of B*27:09/photoRL9 (gray, PDB 7ALO) and B*27:09/RL9 (cyan, PDB 1OF2), superimposed based on the heavy chain. PhotoRL9 is shown in orange to address its orientation different from RL9. The overall structure of B*27:09/photoRL9 was nearly identical to the structure of B*27:09/RL9 and shows the typical hallmarks of a stable peptide-MHC-I (pMHC-I) complex. **c)** Close view of F-pocket region with H-bonds (green dashed line) in the structure of B*27:09/photoRL9. Missing H-bond in B*27:09/photoRL9 is colored in red. Residues in B*27:09/RL9 showing different side-chain orientation is colored cyan. L9 is shown in orange. **d)** Top view of the binding groove with the bound peptide shown in stick in the structure of B*27:09/photoRL9 (gray). Residues showing different side-chain orientation compared to B*27:09/RL9 (cyan) are labelled as sticks.

Supplementary Figure 7

a

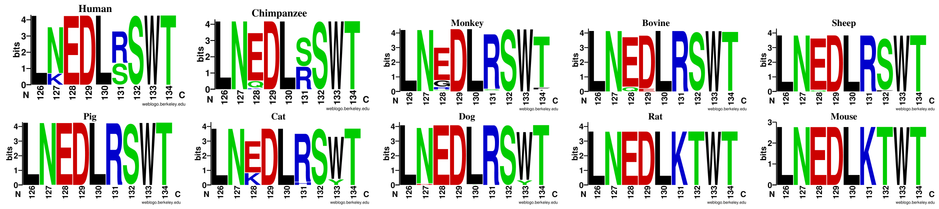

b

```
                                              187        196
SP|O15533|TPSN_HUMAN               PPPFGLEWRRQHLGKGHLLLAATPGLNGQMPAAQEGAV 236
TR|K7CQY4|K7CQY4_PANTR             PPPFGLEWRRQHLGKGHLLLAATPGLNGQMPAAQEGAV 236
TR|A0A2K5U9W2|A0A2K5U9W2_MACFA     PPPFGLEWRRQHLGKGHLLLAATPGLNGQMPAAQEGAV 236
TR|F7H7T7|F7H7T7_MACMU             PPPFGLEWRRQHLGKGHLLLAATPGLNGQMPAAQEGAV 236
SP|Q5TJE4|TPSN_CANLF               PPPFGLEWRRQHLGKGHLLLAATPGLHEQMPAAQDGAV 236
TR|F6T753|F6T753_HORSE             PPPFGLEWRRQHLGKGHLVLAATPGLAAQMPAAQDGAV 236
TR|M3W1E5|M3W1E5_FELCA             PPPFGLEWRRQHLGKGHLLLAATPGLSEQMPPAQEGAV 236
TR|W5PI11|W5PI11_SHEEP             PPPFGLEWRRQHLGKGHLLLAVTPGLNGQVPAAREGAV 230
TR|A0A4X1TAH3|PIG                  PPPFGLEWRRQHLGKGHLLLAATPGLSGQVPAAREGAV 237
TR|Q2NL04|Q2NL04_BOVIN             PPPFGLEWRRQHLGKGHLLLAVTPGLNGQVPAAREGAV 235
TR|M3XTF6|M3XTF6_MUSPF             PPPFGLEWRHQHLGKGHLLLAATPGLCEQMPTPQEGAV 236
TR|G1L3Z3|G1L3Z3_AILME             PPPFGLEWRRQHLGKGHLLLAATPGLGEQMPAAQEGTV 236
TR|A0A384AHY2|A0A384AHY2_BALAS     PPPFGLEWRRQHLGKGHLMLAATPGLSGQMPVAREGAV 236
TR|A0A2U3WGD7|A0A2U3WGD7_ODORO     PPPFGLEWRRQHLGKGHLLLAATPGLGEQMPAAQEGAV 236
TR|Q99JC6|Q99JC6_RAT               PPPFGLEWRRQHRGKGHLLLAAIPGLLEKMPPAREKAV 237
SP|Q9R233|TPSN_MOUSE               PPPFGLEWRRQHRGKGHLLLAATPGLAGRMPPAQEKAT 239
                                   *********:** *****:**. ***  : *  :: :.
```

Supplementary Figure 7. The loop$_{126-133}$ of heavy chain and loop$_{187-196}$ of tapasin among species is conserved. a) Sequence logos of residues 126-134 of MHC-I of different species. The loop$_{126-133}$ is highly conserved within and between species. Sequence logos were generated in https://weblogo.berkeley.edu. T134 was included because it has been shown of importance for PLC complex assembly and is next to the loop$_{126-133}$. **b)** Sequence alignment of residues 179-236 of tapasin from different species. Species in bold are the ones displayed in supplementary Fig. 8. Residues in human tapasin showing impaired MHC-I binding in previous studies are highlighted green. Amino acids at number 187 and 196 were noted to indicate the beginning and end of the loop$_{187-196}$ which interacts with the highly conserved loop$_{126-133}$ of MHC-I as shown in Supplementary Fig. 2c.

Supplementary Figure 8

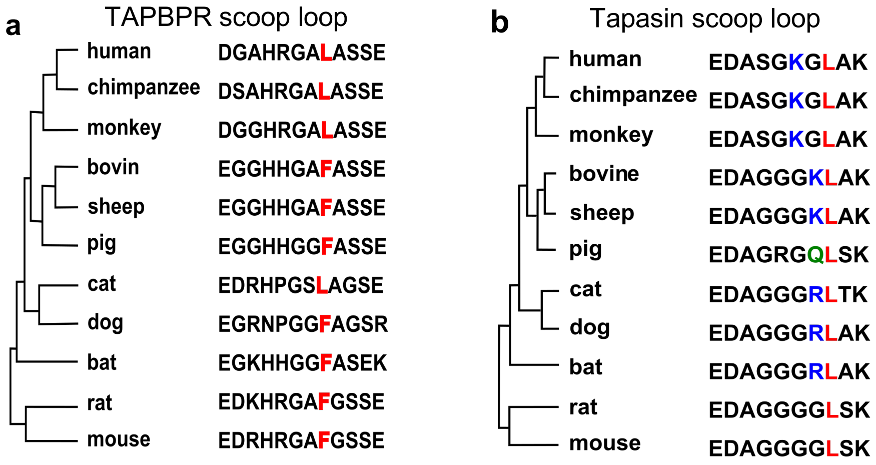

Supplementary Figure 8. TAPBPR and Tapasin scoop loop sequences from different species. a) Evolution tree of TAPBPR from common species and the alignment of corresponding scoop loop sequence. The critical residues are colored in red. **b)** Evolution tree of tapasin from common species and the alignment of corresponding scoop loop sequence. Basic residue (K and R) is highlighted in blue, L is highlighted in red, and polar residue (Q) is highlighted in olive.

Supplementary Figure 9

a

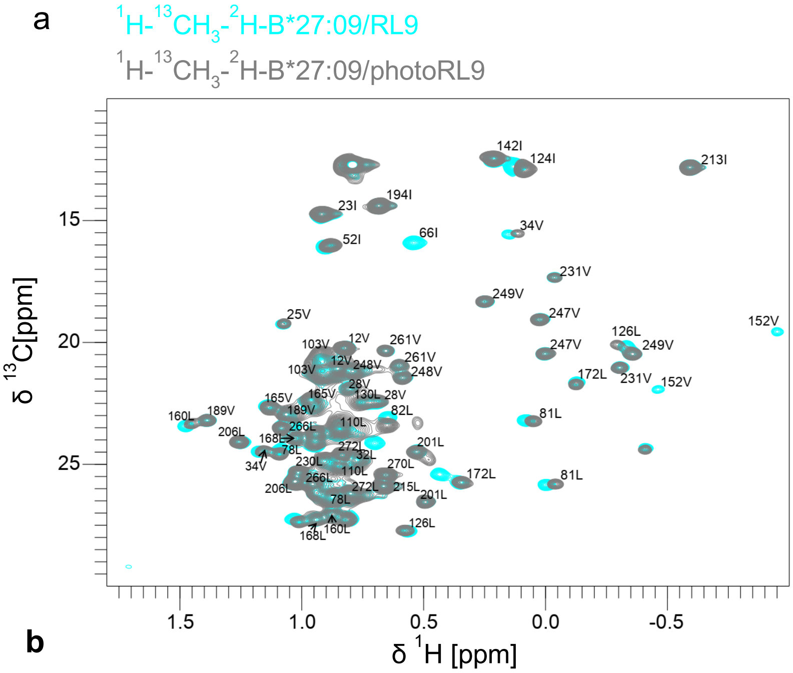

b

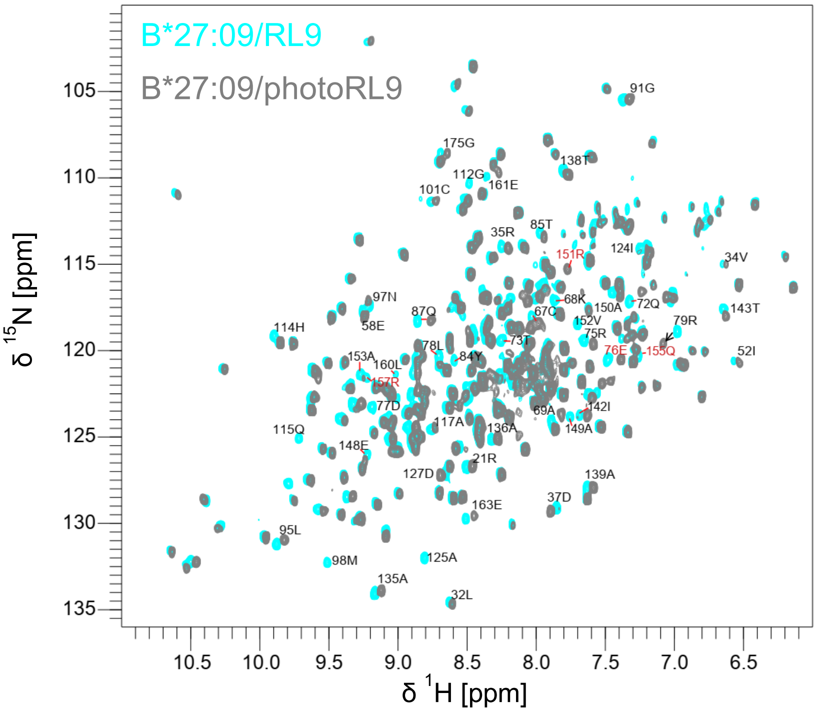

Supplementary Figure 9. The assignments of ILV methyl group of B*27:09/photoRL9 and the assignments of B*27:09/photoRL9. a) Overlay of ^{1}H-^{13}C HSQC spectra of B*27:09/RL9 (cyan, 150 μM) and B*27:09/photoRL9 (grey, 170 μM) with assignments, measured in PBS 7.2 buffer with 10% D_2O at 300K on a Bruker Avance III 700 MHz spectrometer. **b)** Overlay of ^{1}H-^{15}N TROSY-HSQC spectra of B*27:09/RL9 (cyan) and B*27:09/photoRL9 (grey). Residues showing different chemical shift were labelled in black. Residues showing different orientation in crystal structure in Supplementary Fig. 6 **d)** are labelled in red.

Supplementary Table 1.

List of peptides used in this paper

Peptide name	Allotype	Sequence	* Predicted affinity (nM)
photoKV9	A*02:01	KILGFVFJ*V	2.08
photoKK9	A*03:01	KLIETYFJ*K	17.74
photoRL9	B*27:05, B*27:09	RRKWRRWJ*L	35.64 for B*27:05<br>196.44 for B*27:09
photoIF9	B*27:05, B*27:09	IRAAPPPJ*F	720.18 for B*27:05<br>1762.61 for B*27:09
FITC-GV9	A*02:01	GILGK^{FITC}VFTV	5.95
FITC-KV9	A*02:01 and F-pocket mutants of A*02:01/A*03:01	KILGK^{FITC}VFTV	4.14 for A*02:01
FITC-RL9	B*27:05, B*27:09	RRKWK^{FITC}RWHL	72.27 for B*27:05<br>476.51 for B*27:09
FITC-IF9	B*27:05, B*27:09	IRAAK^{FITC}PPLF	385.52 for B*27:05<br>2143.59 for B*27:09
FITC-KK9	A*03:01	KLIEK^{FITC}YFSK	18.04
FITC-KV9-2	A*02:01 and F-pocket mutants of A*02:01 / A*03:01	KLIEK^{FITC}YFSV	2.19 for A*02:01<br>5234.98 for A*03:01
KV9	A*02:01	KILGFVFTV	2.31
KK9	A*03:01	KLIETYFSK	21.68
RL9	B*27:05, B*27:09	RRKWRRWHL	50.24 for B*27:05<br>322.6 for B*27:09
IF9	B*27:05, B*27:09	IRAAPPPLF	479.27 for B*27:05<br>1589.47 for B*27:09

*affinity values were predicted by NetMHCpan4.1 Server. In the manuscript we consider peptide with affinities < 500 nM has high affinity, while those between 500 nM and 2 μM are described as medium affinity.

Supplementary Table 2.

List of melting temperature of pMHC-I complex

Allotype	Refolded peptide	T_m before UV (°C)	T_m after UV (°C)
A*02:01	photoKV9	51.41 ± 1.27	37.81 ± 0.57
A*03:01	photoKK9	51.43 ± 0.79	50.90 ± 0.05
B*27:09	photoRL9	54.55 ± 0.13	49.61 ± 0.04
	photoIF9	57.94 ± 0.18	43.74 ± 0.37
	IF9	56.2 ± 0.13	
	RL9	60.2 ± 0.18	
B*27:05	photoRL9	57.58 ± 0.08	51.31 ± 0.14
	photoIF9	51.13 ± 0.21	49.28 ± 0.34
	RL9	61.88 ± 1.29	

Supplementary Table 3.

List of designed F-pocket and loop$_{126\text{-}133}$ mutants in this study

Allotype	mutation	Refolded peptide	note
A*02:01	Y116D	photoKV9	
	Y116D	photoKK9	
	H114D	photoKV9	
	H114R/Y116D	photoKV9	refolding failed
	H114R/Y116D	photoKK9	refolding failed
A*03:01	D116Y	photoKK9	
	D116Y	photoKV9	
	R114H/D116Y	photoKK9	
	R114H/D116Y	photoKV9	
B*27:05	D116Y	photoRL9	
	H114D	photoRL9	refolding failed
	H114R	photoRL9	

Supplementary Table 4.

Primers used in this study are listed below

Mutant	Forward	Reverse
A*02:01H114D	cttcctccgcgggtacGACcagtacgcctacgac	gtcgtaggcgtactgGTCgtacccgcggaggaag
A*02:01Y116D	cgcgggtaccaccagGACgcctacgacggcaag	cttgccgtcgtaggcGTCctggtggtacccgcg
A*02:01H114R/Y116D	ttcctccgcgggtacAGAcagGACgcctacgacggcaag	ttcctccgcgggtacAGAcagGACgcctacgacggcaag
A*03:01D116Y	cgtggttatcgtcagTATgcctatgatggtaaag	ctttaccatcataggcATActgacgataaccacg
A*03:01R114H/D116Y	tttctgcgtggttatCATcagTATgcctatgatggtaaag	ctttaccatcataggcATActgATGataaccacgcagaaa
B*27:05H114D	CTCCTCCGCGGGTACGACCAGGACGCCTACGAC	GTCGTAGGCGTCCTGGTCGTACCCGCGGAGGAG
B*27:05H114R	CTCCTCCGCGGGTACAGGCAGGACGCCTACGAC	GTCGTAGGCGTCCTGAGGGTACCCGCGGAGGAG
B*27:05D116Y	CCGCGGGTACCACCAGTACGCCTACGACGGCAAG	CTTGCCGTCGTAGGCGTACTGGTGGTACCCGCGG
ERp57-Tsn^{K16G}	gaggacgcttccggcGGAggccttgctaaacgtc	gacgtttagcaaggccTCCgccggaagcgtcctc
ERp57-Tsn^{K16L}	gaggacgcttccggcCTGggccttgctaaacgtc	gacgtttagcaaggccCAGgccggaagcgtcctc
ERp57-Tsn^{L18G}	gcttccggcaagggcGGAgctaaacgtcctgg	ccaggacgtttagcTCCgcccttgccggaagc
ERp57-Tsn^{L18K}	gcttccggcaagggcAAAgctaaacgtcctggtg	caccaggacgtttagcTTTgcccttgccggaagc
ERp57-Tsn^{GGLGL}	ttcgttgaggacgctGGAggcCTGggccttgcta	gcaaggccCAGgccTCCagcgtcctcaacgaac
*ERp57-Tsn^{GGGGG}	gctGGAggcGGAggcGGAgctaaacgtcctggtg	caccaggacgtttagcTCCgccTCCgccTCCagc
*ERp57-Tsn^{GGGGL}	ttcgttgaggacgctGGAggcGGAggccttgcta	gcaaggccTCCgccTCCagcgtcctcaacgaac
*ERp57-Tsn^{GGGGK}	gctGGAggcGGAggcAAGgctaaacgtcctggtg	caccaggacgtttagcCTTgccTCCgccTCCagc
*ERp57-Tsn^{GGLGG}	gctGGAggcCTGggcGGAgctaaacgtcctgg	ccaggacgtttagcTCCgccCAGgccTCCagc
*ERp57-Tsn^{GGKGG}	gctGGAggcAAGggcGGAgctaaacgtcctgg	gacgtttagcTCCgccCTTgccTCCagcgtcctc

Mutates were generated by using WT as template unless otherwise stated.

* ERp57-Tsn^{GGGGL} was mutated by using ERp57-Tsn^{K16G} as templat. ERp57-Tsn^{GGGGG} and ERp57-Tsn^{GGGGK} were mutated by using ERp57-Tsn^{GGGGL} as template. ERp57-Tsn^{GGLGG} and ERp57-Tsn^{GGKGG} were mutated by using ERp57-Tsn^{GGLGL} as template.

Supplementary Table 5.

Data collection and refinement statistics.

**Data collection**	
Wavelength [Å] [a]	0.91841
Resolution [Å] [a]	50.00 - 1.80 (1.92 - 1.80)
Space group	*P*1
Unit cell a;b;c [Å]	45.2; 69.6; 82.6;
α; β, γ [°]	80.2; 88.3; 89.9
Total reflections [a]	333,000 (59,466)
Unique reflections [a]	89,225 (15,619)
Multiplicity [a]	3.7 (3.8)
Completeness [%] [a]	97.1 (96.8)
<I/σ(I)> [a]	5.4 (1.06)
Wilson B-factor	32.2
R_{meas} [%] [a, b]	19.8 (161.9)
$CC_{1/2}$ [c]	99.0 (45.0)
**Refinement**	
Resolution [Å] [a]	50.00 - 1.80 (1.82 - 1.80)
Test set [%]	5.0
Non-hydrogen atoms	7346
R_{work} [%] [a, d]	22.3 (38.1)
R_{free} [%] [a, f]	26.5 (39.9)
Asymmetric unit	
Heavy chain (residues)	276 (chain A) / 276 (chain D)
□$_2$m (residues)	99 (chain B) / 99 (chain E)
peptide (residues)	9 (chain C) / 9 (chain F)
Ligand molecules (PEG3350, glycerol, Tris)	17
water molecules	527
Average B-factor [Å^2]	34.3
heavy chain	32.0 (chain A) / 35.1 (chain D)
□$_2$m	32.8 (chain B) / 33.4 (chain E)
peptide	44.9 (chain C) / 44.9 (chain F)
ligands (PEG3350, glycerol, Tris)	51.7
water molecules	36.4
r.m.s.d. [g] (bonds) [Å]	0.006
r.m.s.d. [g] (angles) [°]	1.032
**Validation**	
Ramachandran favoured [%]	96.8
Ramachandran allowed [%]	3.2
Ramachandran outliers [%]	0.0
Rotamer outliers [%]	1.27
Molprobity score [i]	1.55
Molprobity clash score [i]	5.47

[a] Values in parentheses refer to the highest resolution shells.

[b] $R_{meas}(I) = \sum_h [N/(N-1)]^{1/2} \sum_i | I_{ih} - <I_h> | / \sum_h \sum_i I_{ih}$, in which $<I_h>$ is the mean intensity of symmetry-equivalent reflections h, I_{ih} is the intensity of a particular observation of h and N is the number of redundant observations of reflection h[63].

[c] $CC_{1/2} = (<I^2> - <I>^2) / (<I^2> - <I>^2) + \sigma^2_\varepsilon$, in which σ^2_ε is the mean error within a half-dataset[64].

[e] $R_{work} = \sum_h | F_o - F_c | / \sum F_o$ (working set, no σ cut-off applied).

[f] R_{free} is the same as R_{work} but calculated on the test set of reflections excluded from refinement.

[g] r.m.s.d. – root-mean-square deviation.

[i] Calculated with MolProbity[59].
