# Supplementary material for: Exchange catalysis by tapasin exploits conserved and allele-specific features of MHC-I molecules
Source: Nat Commun. 2021 Jul 9;12:4236. doi: 10.1038/s41467-021-24401-4 (PMC8271027; doi:10.1038/s41467-021-24401-4)
Supplement: Supplementary file 2 — Reporting Summary [file 41467_2021_24401_MOESM2_ESM.pdf]

nature research

Corresponding author(s): Christian Freund

Last updated by author(s): Mar 24, 2021

Reporting Summary

Nature Research wishes to improve the reproducibility of the work that we publish. This form provides structure for consistency and transparency in reporting. For further information on Nature Research policies, see our Editorial Policies and the Editorial Policy Checklist.

Statistics

For all statistical analyses, confirm that the following items are present in the figure legend, table legend, main text, or Methods section.

n/a	Confirmed	
	x	The exact sample size (n) for each experimental group/condition, given as a discrete number and unit of measurement
	x	A statement on whether measurements were taken from distinct samples or whether the same sample was measured repeatedly
	x	The statistical test(s) used AND whether they are one- or two-sided<br>*Only common tests should be described solely by name; describe more complex techniques in the Methods section.*
x		A description of all covariates tested
x		A description of any assumptions or corrections, such as tests of normality and adjustment for multiple comparisons
	x	A full description of the statistical parameters including central tendency (e.g. means) or other basic estimates (e.g. regression coefficient) AND variation (e.g. standard deviation) or associated estimates of uncertainty (e.g. confidence intervals)
	x	For null hypothesis testing, the test statistic (e.g. F, t, r) with confidence intervals, effect sizes, degrees of freedom and P value noted<br>*Give P values as exact values whenever suitable.*
x		For Bayesian analysis, information on the choice of priors and Markov chain Monte Carlo settings
x		For hierarchical and complex designs, identification of the appropriate level for tests and full reporting of outcomes
x		Estimates of effect sizes (e.g. Cohen's d, Pearson's r), indicating how they were calculated

Our web collection on statistics for biologists contains articles on many of the points above.

Software and code

Policy information about availability of computer code

Data collection	TopSpin3.2, PerkinElmer VICTOR3V, XDS.
Data analysis	CcpNmr Analysis 2.4.2, OriginPro 2019, PyMol, Phaser, Phenix, COOT, MOLPROBITY

For manuscripts utilizing custom algorithms or software that are central to the research but not yet described in published literature, software must be made available to editors and reviewers. We strongly encourage code deposition in a community repository (e.g. GitHub). See the Nature Research guidelines for submitting code & software for further information.

Data

Policy information about availability of data

All manuscripts must include a data availability statement. This statement should provide the following information, where applicable:

- Accession codes, unique identifiers, or web links for publicly available datasets
- A list of figures that have associated raw data
- A description of any restrictions on data availability

NMR assignments for methyl group of ILV of 27:09 heavy chain have been updated into the Biological Magnetic Resonance Data Bank (http://www.bmrb.wisc.edu) under accession number 25713. The X-ray structure of B*27:09 in complex with photoRL9 is deposited in the RCSB Protein Data Bank with accession codes PDB ID 7ALO. Previously solved structures were obtained from the Protein Data Bank (https://www.rcsb.org/). Peptides used for motif generation were downloaded from IEDB. MHC-I sequences used for analysis were download from https://www.ebi.ac.uk/ipd/imgt/hla/. Source data are provided with this paper. Other data are available from the corresponding authors upon reasonable request.

Field-specific reporting

Please select the one below that is the best fit for your research. If you are not sure, read the appropriate sections before making your selection.

[x] Life sciences [ ] Behavioural & social sciences [ ] Ecological, evolutionary & environmental sciences

For a reference copy of the document with all sections, see nature.com/documents/nr-reporting-summary-flat.pdf

Life sciences study design

All studies must disclose on these points even when the disclosure is negative.

Sample size	Sample size is not applicable to NMR measurements since our data do not report or not depend on quantitative values. For FP assays, experiments were performed with at least three different batches of tapasin mutants or TAPBPR. In each batch three technical repeats were performed to ensure the reproducibility of our results. The repeats of assays are given in the figure legends and are standard for the field.
Data exclusions	no data were excluded from the analysis
Replication	For relative activity of tapasin loop mutants, FP assays were performed at least with three different batches of purified tapasin scoop loop mutants.
Randomization	all experiments were performed randomized.
Blinding	Measurements of mass spectrometric samples were performed by a scientist who was not informed about the experimental setup.

Reporting for specific materials, systems and methods

We require information from authors about some types of materials, experimental systems and methods used in many studies. Here, indicate whether each material, system or method listed is relevant to your study. If you are not sure if a list item applies to your research, read the appropriate section before selecting a response.

Materials & experimental systems

n/a	Involved in the study	
[ ]	[x]	Antibodies
[x]	[ ]	Eukaryotic cell lines
[x]	[ ]	Palaeontology and archaeology
[x]	[ ]	Animals and other organisms
[x]	[ ]	Human research participants
[x]	[ ]	Clinical data
[x]	[ ]	Dual use research of concern

Methods

n/a	Involved in the study	
[x]	[ ]	ChIP-seq
[x]	[ ]	Flow cytometry
[x]	[ ]	MRI-based neuroimaging

Antibodies

Antibodies used	Anti-His-HRP(Miltenyi Biotec, clone: GG11-8F3.5.1)
Validation	Recombinant Antibody is commercially available and binds to common epitope tag. Monoclonal antibody from mouse.
